# Microalgae-Derived Peptides Targeting Lifestyle-Related Diseases: Discovery, Mechanisms, Structure–Activity Relationships, and Structural Modifications

**DOI:** 10.3390/antiox14101170

**Published:** 2025-09-25

**Authors:** Mohammed S. Qoms, Sok Kuan Wong, Norsyahida Mohd Fauzi, Khairana Husain, Suzana Makpol, Jen Kit Tan

**Affiliations:** 1 Department of Biochemistry, Faculty of Medicine, Universiti Kebangsaan Malaysia, Kuala Lumpur 56000, Malaysia; 2 Department of Pharmacology, Faculty of Medicine, Universiti Kebangsaan Malaysia, Kuala Lumpur 56000, Malaysia; 3 Centre for Drug and Herbal Development, Faculty of Pharmacy, Universiti Kebangsaan Malaysia, Kuala Lumpur 50300, Malaysia

**Keywords:** microalgae-derived bioactive peptides, mode of action, structural features, health properties, non-communicable diseases, oxidative-related diseases

## Abstract

Microalgae are an emerging source of bioactive peptides with promising therapeutic potential against lifestyle-related diseases such as oxidative stress-related conditions, hypertension, diabetes, and obesity. While numerous studies have investigated the biological activities of microalgae-derived peptides, a comprehensive understanding of their structural features and structure–activity relationships remains limited. This review provides a detailed overview of current strategies used to discover bioactive peptides from microalgae, encompassing both conventional and computational approaches. Particular emphasis is placed on correlating identified peptide sequences with their reported biological activities to provide critical insights into the key structural motifs responsible for activity. Furthermore, recent advances in peptide modification techniques are discussed in the context of enhancing the bioactivity of microalgae-derived peptides. By integrating discovery strategies, structure–activity relationships, and emerging trends in peptide modification, this review highlights the potential of microalgae-derived peptides as next-generation therapeutic agents for managing lifestyle-related diseases and identifies opportunities for future research and clinical translation.

## 1. Introduction

Globally, non-communicable diseases have become a major health concern in recent decades, often attributed to a combination of genetic, metabolic, and lifestyle factors [1]. According to the World Health Organization (WHO), noncommunicable diseases are the primary cause of global mortality, accounting for approximately 75% of non-pandemic-related deaths in 2021, with cardiovascular diseases causing at least 19 million deaths, cancers (10 million), chronic respiratory diseases (4 million), and diabetes (1.6 million) [1]. While synthetic compounds or drugs offer promising therapeutic strategies for managing chronic diseases such as hyperlipidaemia, diabetes, and hypertension and mitigating their complications, their long-term use can lead to various side effects such as hepatotoxicity, myopathy, gastrointestinal disturbances, and renal impairment [2,3,4,5]. Consequently, food and pharmaceutical industries, as well as consumers, are demanding products free from synthetic substances. To fulfil this demand, innovative approaches such as the use of natural bioactive compounds have gained more attention, particularly natural bioactive peptides [6,7].

Bioactive peptides are short chains of amino acids consisting of 2–50 residues with low molecular weight [8]. Depending on their structural and physicochemical characteristics, bioactive peptides can possess single or multiple pharmacological effects, such as antioxidant, antihypertensive, anti-obesity, antidiabetic, anti-ageing, or antimicrobial [9,10,11]. Conventionally, bioactive peptides are generated from parent proteins using different methods such as enzymatic hydrolysis, microbial fermentation, or combined enzymatic-fermentation hydrolysis [12,13,14,15]. Recently, the discovery of bioactive peptides has been advanced by combining conventional techniques with computer-based approaches (in silico), which has led to the development of novel bioactive peptides with strong activity [16,17]. Production of bioactive peptides by any means of hydrolysis methods typically yields a crude peptide mixture, referred to as protein hydrolysates, with varying structural and physicochemical properties [18,19,20]. Therefore, bioactive peptides require further fractionation and purification steps, which usually involve membrane filtration techniques (e.g., ultrafiltration) and chromatographic techniques (e.g., gel permeation, size exclusion, and ion exchange), followed by identifying the bioactive sequences using chromatography-mass spectrometry techniques (e.g., LC-MS/MS) [21,22].

Research on natural bioactive peptides derived from algae [23], plants [21], and animal [9] protein sources, as well as those naturally produced by microorganisms [10], is constantly evolving. Compared to conventional plant and animal protein sources, aquatic microalgae are a promising alternative protein source for generating natural bioactive peptides, as reflected by the increasing number of publications in the past decade, due to their rapid-growing nature that is driven by excellent ecological adaptation, high protein content, and favourable amino acid profiles. Additionally, numerous microalgae species such as *Arthrospira platensis* (spirulina), *Chlorella vulgaris*, *Auxenochlorella protothecoides*, *Haematococcus pluvialis*, *Chlamydomonas reinhardtii*, and *Dunaliella bardawil* have been granted GRAS (generally recognised as safe) and approved by the Food and Agriculture Organisation (FAO) as food/food ingredients [24]. A recent review by Kumar et al. [25] observed that microalgae biomasses were found to accumulate high protein content ranging between 23 and 63%, with the most studied microalgae proteins being phycobiliproteins, phycocyanin, and phycoerythrin. Additionally, most microalgae species were shown to have a balanced amino acid profile containing both essential and non-essential amino acids, with the total essential amino acids being very close to or even exceeding those of reference proteins (e.g., soybean and egg) and the recommended amount by FAO/WHO [24]. The high protein content and complete amino acid profile of microalgae proteins make them a great source of biologically active peptides with potential in managing and preventing chronic diseases owing to their multiple pharmacological properties, including but not limited to antioxidant [26], antihypertensive [27], antidiabetic [28,29], anti-ageing [30], anti-inflammatory [31], antimicrobial [32], as well as gut modulatory effects [33]. Nevertheless, their wide application in food and pharmaceutical industries is still limited as some peptides encounter a few challenges, such as susceptibility to enzymatic cleavage and low stability under different processing conditions (e.g., harsh acidic/alkaline pH and high temperature) [34,35]. These limitations have driven researchers to explore novel discovery approaches and better understand their structure–activity relationship that can help in peptide modification and in maximising their activity and stability, thus bridging the gap to their real-life application.

A few reviews on microalgae-derived peptides have been published over the past decade. For example, Fan et al. [36] provided a broad overview of algae-derived peptides with emphasis on their general bioactivities, while Jiang et al. [37] presented a mini-review highlighting the antihypertensive potential of microalgae ACE-inhibitory peptides, including discussions of their inhibition patterns, molecular docking, and related antioxidant and anti-inflammatory pathways. Karabulut et al. [38] critically assessed algae proteins and peptides from the perspective of extraction strategies, technological properties, and commercial applications, with some reference to biofunctional activities, whereas Ashraf et al. [39] focused on bioactivities of microalgae peptides in the context of functional food innovations and regulatory aspects. However, despite these valuable contributions, there remains a lack of comprehensive synthesis that integrates mechanistic insights of microalgae-derived peptides across diverse bioactivities with a critical evaluation of their structural features to better understand structure–activity relationships and strategies to enhance bioactivities. Therefore, this review provides an in-depth overview of the discovery of microalgae-derived bioactive peptides (conventional and computational approaches) and their corresponding mechanisms. It further emphasises structure–activity relationships by critically analysing the structural features of identified microalgae bioactive peptide sequences with known effective concentrations and confirmed in vitro/in vivo activity. In addition, recent trends in structural modification strategies are discussed, highlighting approaches that can lead to the discovery of potent and stable microalgae-derived bioactive peptides.

## 2. Preparation and Discovery of Microalgae-Derived Protein Hydrolysates/Bioactive Peptides

Preliminary processing targeting microalgae cell disruption and protein extraction is often applied prior to the generation of protein hydrolysates/peptides. Several conventional techniques, such as freezing-thawing, drying, bead-milling, homogenisation, and alkaline treatment, have been proposed for different species of microalgae. Recently, researchers have also introduced innovative green techniques such as microwave, ultrasound, high-pressure, pulsed electric field, supercritical water extraction, and deep eutectic solvents [24,40,41,42].

### 2.1. Enzymatic Hydrolysis

**Figure 1:**
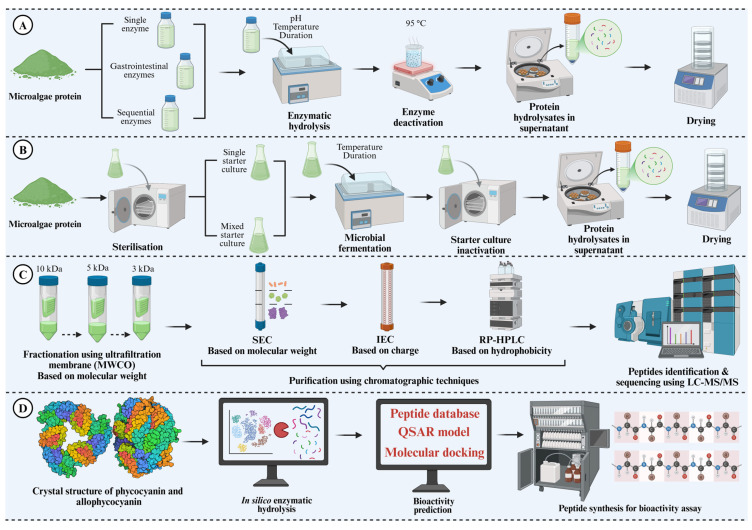
Schematic representation of the overall workflow for obtaining bioactive peptides from microalgae proteins, including preparation, purification and discovery steps: (**A**) enzymatic hydrolysis, (**B**) microbial fermentation, (**C**) peptide purification and identification, and (**D**) bioinformatics and computational tools. Created in BioRender. Qoms, M. (2025) https://BioRender.com/6tw09tw, accessed on 21 August 2025.

Enzymatic hydrolysis is one of the most widely employed approaches for generating protein hydrolysates/bioactive peptides with different health-promoting effects (Figure 1A). The enzymatic process involves the controlled breakdown of large proteins into smaller peptides through the action of specific proteases. The resulting peptides possess several remarkable bioactivities that make them highly valuable ingredients in the food and pharmaceutical industries [16]. The extent to which a protein substrate is cleaved into smaller peptides is determined by the degree of hydrolysis (DH), which directly influences several key aspects (structure and function) of protein hydrolysates. Generally, the extent of protein hydrolysis is highly dependent on several parameters, such as the protein substrate, the type of protease used for substrate hydrolysis and the hydrolysis, duration [16,43]. Several methods, including o-phthaldialdehyde (OPA), trinitrobenzenesulfonic acid (TNBS), and pH-stat, have been reported in the literature for monitoring the DH during proteolysis and peptide generation [44]. Commercial proteases from different sources have been widely used for the production of bioactive peptides, as follows: (i) microbial sources such as alcalase, flavourzyme, neutrase, protamex, and umamizyme; (ii) plant sources such as papain, bromelain, and ficin; and (iii) animal sources such as pepsin, trypsin, and α-chymotrypsin [45].

Proteases are highly specific enzymes that recognise and bind to specific amino acid sequences or residues (cleavage sites) within a protein molecule [44]. Proteolytic enzymes can efficiently and rapidly break protein substrates into shorter amino acid fragments, thus generating protein hydrolysates with high peptide yield compared to other production methods. Due to their specificity, enzymatic hydrolysis is a reproducible method and can generate consistent products. Furthermore, enzymatic hydrolysis is performed under mild conditions, making it an environmentally friendly process, as no toxic substances or undesirable by-products are generated in the final products, unlike chemical hydrolysis [44,45]. Such eco-friendly aspects of enzymatic hydrolysis align with sustainability goals and are particularly advantageous in the food and pharmaceutical industries, where the production of safe and clean-label products is of interest. However, enzymatic hydrolysis is an expensive process due to the cost of enzymes. Therefore, immobilised enzyme techniques have been introduced for the production of bioactive peptides, where the enzymes can be recovered and reused after hydrolysis. This technique consists of a two-phase system, in which one phase contains the enzyme and the other phase contains the product [16]. Another disadvantage is over-hydrolysis, which occurs when process conditions are not properly controlled, leading to the production of bitter peptides [46]. Thus, the hydrolysis conditions must be carefully controlled to produce bioactive peptides with high and desired quality.

Several bioactive protein hydrolysates and peptides have been produced from microalgae proteins through enzymatic hydrolysis using different proteases. The most frequently investigated bioactivities of microalgae-derived peptides are antioxidant and antihypertensive effects. In this context, López-Rodríguez et al. [47] compared the production of antioxidant protein hydrolysates from two different species of *Arthrospira*, namely *Arthrospira maxima* and *Arthrospira platensis*, using papain. Both species showed similar DH of 22.40% and 22.88%, respectively; however, *A. maxima* demonstrated stronger ABTS radical scavenging activity than *A. platensis* (87.47% vs. 75.91%). Gharehbeglou et al. [12] studied the antioxidant potential of *Chlorella* sp. hydrolysed with alcalase, pancreatin, trypsin, and pepsin, resulting in a DH of 32.20%, 34.10%, 37.83%, and 26.73%. The antioxidant activity varied based on the enzyme type and assay employed, with alcalase producing the most active peptides among the enzymes. The alcalase-generated hydrolysates exhibited the highest ferric reducing antioxidant power (FRAP), copper chelating, DPPH, nitric oxide (NO), and hydroxyl radical scavenging activities. Pancreatin exhibited the highest ABTS radical scavenging activity and FRAP, while trypsin generated the strongest iron chelating capacity. In another study reported by Wang et al. [48], six different enzymes (pepsin, trypsin, papain, alcalase, neutrase, and flavourzyme) were employed to hydrolyse *A. platensis* protein into bioactive peptides with antioxidant and antihypertensive effects. The bioactivities of the generated hydrolysates were influenced by the enzyme type and the duration of hydrolysis, whereby hydrolysis using alcalase for 120 min generated the highest antioxidant activity measured by iron-chelating capacity, ABTS, and hydroxyl radical scavenging assays, while trypsin hydrolysis for 240 min produced the most active ACE inhibitory protein hydrolysates. Recently, a research group investigated the potential use of extracellular protease produced by the marine bacterium *Pseudoalteromonas* sp. JS4-1 to generate antioxidant peptides from *Chlorella protothecoides* and *S. platensis*. The resulting hydrolysates from both microalgae exhibited good scavenging activity against DPPH, hydroxyl, and hydrogen peroxide radical scavenging activities [49,50]. In another study on different *Chlorella* species, namely *Chlorella sorokiniana*, protease N produced the most potent in vitro ACE-inhibitory protein hydrolysates, with an IC_50_ of 0.035 mg/mL and a higher peptide content compared to those generated using protamex. Also, the *C. sorokiniana* protein hydrolysates produced with protease N demonstrated a promising in vivo antihypertensive activity in spontaneously hypertensive rats, reducing both systolic and diastolic blood pressure after 6 h of oral administration at doses of 30 and 60 mg peptide/kg body weight [51]. Li et al. [52] compared the effectiveness of two different proteases, pepsin and trypsin, to generate hypotensive and hypoglycaemic peptides from *Chlorella pyrenoidosa*. The trypsin-generated protein hydrolysates showed a higher DH of 35.50% compared to pepsin, which showed almost half of that value. However, both protein hydrolysates exhibited comparable in vitro antihypertensive ACE inhibitory activity (84.20 and 78.60%) when tested at 1 mg/mL. Additionally, they showed antidiabetic potential by inhibiting DPP-IV activity (63.70 and 69.60%) at a higher concentration of 5 mg/mL. Furthermore, the pepsin- and trypsin-generated protein hydrolysates exhibited good hypotensive (61.50–69.90%) and hypoglycaemic (38.40–42.50%) effects in a Caco-2 cell model at 5 mg/mL.

In addition to antioxidant, antihypertensive, and antidiabetic activities, microalgae protein hydrolysates have demonstrated potent anticancer effects against different cancer cells. Wang and Zhang [53] investigated the efficacy of three different proteases (trypsin, alcalase, and papain) on the antiproliferative effect of *S. platensis* protein hydrolysates at 500 µg/mL on five different cancer cells (HepG-2, MCF-7, SGC-7901, A549, and HT-29). They reported that alcalase-generated hydrolysate was the most effective against all the cancer cell lines tested, with inhibition ranging between 81.20 and 95.88%, followed by trypsin (74.00–88.00%) and papain (73.61–96.11%). These results were closely correlated with the DH, where alcalase produced the highest DH of 34.39%, followed by trypsin (18.26%) and papain (11.12%). Interestingly, the alcalase-generated hydrolysates demonstrated higher anti-tumour activity than the standard drug 5-flurouracil (5-FU), while the trypsin- and papain-produced hydrolysates were comparable in effect to 5-FU.

The anti-obesity potential of microalgae protein hydrolysates and peptides has been reported by several research groups [54,55,56,57]. For instance, enzymatic hydrolysis of *S. platensis* was investigated using various proteases, including papain, alcalase, trypsin, pepsin, and protamex. All the generated hydrolysates possessed anti-obesity effects by inhibiting the activity of pancreatic lipase in vitro. It was observed that the highest pancreatic lipase-inhibitory effect was noted for protamex (66.23%), followed by alcalase (51.29%), pepsin (50.61%), trypsin (30.65%), and papain (27.24%), with a DH of 34.10%, 20.80%, 3.60%, 20.20%, and 18.30%, respectively [57]. For *C. pyrenoidosa*, enzymatic hydrolysis using different proteases showed that alcalase hydrolysates (IC_50_ = 87.79 µg/mL) were the most active in inhibiting the pancreatic lipase, compared to pepsin, trypsin, and papain [56].

Production of bioactive peptides using the sequential gastrointestinal digestion (SGID) approach has emerged as an effective alternative to conventional enzymatic hydrolysis using a single enzyme. SGID mimics the physiological conditions (enzymes, pH, salts, and duration) of the human gastrointestinal tract. This model possesses several advantages, including rapid processing, cost effectiveness, and the fact that it bypasses the need for ethical approval [58]. Furthermore, protein substrates can be cleaved at different sites by the action of sequential hydrolysis involving multiple gastrointestinal proteases, generating bioactive peptides with potent activities and distinct characteristics. Chen et al. [59] compared the effectiveness of SGID and single-enzyme hydrolysis for the production of bioactive peptides from *Isochrysis zhanjiangensis*. They observed that SGID using pepsin, trypsin, and chymotrypsin produced stronger antioxidant peptides (as measured by DPPH, hydroxyl, superoxide, and alkyl radical scavenging activity) than the use of individual gastrointestinal enzymes. In another study conducted by Paterson et al. [60], SGID based on the INFOGEST model (an internationally standardised protocol for SGID) was used to hydrolyse two different microalgae, namely *C. vulgaris* and *Tetraselmis chuii*. The digested microalgae samples showed strong antioxidant activity, as determined by ABTS and DPPH radical scavenging, FRAP, and oxygen radical antioxidant capacity (ORAC). The same research group also enzymatically hydrolysed *Nannochloropsis gaditana* using the INFOGEST model and found that the digested sample positively modulated human gut microbiota and their secondary metabolites, such as short-chain fatty acids [33].

In summary, it is evident that several factors, such as microalgae species (protein substrate), protease type, hydrolysis duration, and DH, play a crucial role in determining the bioactivities of enzymatically hydrolysed bioactive protein hydrolysates/peptides. A general trend observed is that a longer hydrolysis period leads to a higher DH, generating low-molecular-weight peptides that often exhibit enhanced bioactivity. However, some studies have reported an opposite trend regarding the DH of the produced protein hydrolysates, suggesting that excessive hydrolysis may lead to the loss of bioactivities. Therefore, selecting an appropriate enzyme and monitoring the bioactivity of interest during protein hydrolysis are crucial for achieving maximum activity. This approach, however, is time-consuming and results in numerous samples that need to be evaluated. To address this challenge, optimisation studies employing mathematical models such as response surface methodology (RSM) are often utilised to minimise experimental runs while maximising the production of potent bioactive peptides with desired activity. A recent study by Amiri et al. [61] employed RSM with central composite design (CCD) to optimise the enzymatic hydrolysis conditions (enzyme concentration and hydrolysis duration) for *Scenedesmus obliquus* protein using alcalase and trypsin, while maintaining the optimum pH and temperature for each enzyme. The goal was to maximise the DH in a shorter hydrolysis duration, based on the hypothesis that higher DH could produce short bioactive peptides with strong biological activity. The experimental model for both enzymes successfully predicted the optimal enzymatic hydrolysis conditions for *S. obliquus* protein with high DH, displaying strong antioxidant (DPPH radical scavenging activity) ranging from 50 to 80%. Cunha et al. [62,63] also utilised RSM with Box–Behnken design (BBD) and successfully optimised the subtilisin hydrolysis conditions (subtilisin concentration, hydrolysis duration, and temperature) of *Chlorella vulgaris* and *Nannochloropsis oceanica* proteins, generating protein hydrolysates with high protein content and potent antioxidant (ORAC) and antihypertensive (ACE inhibitory) activities.

### 2.2. Microbial Fermentation

The microbial fermentation technique involves the use of protease-producing microorganisms that can hydrolyse protein substrates into smaller bioactive peptides with multifunctional and biological properties (Figure 1B). Fermentation has been widely used to hydrolyse and produce protein hydrolysates from different protein sources such as oilseed meal, soybean, okara waste, and faba bean [64,65]. Typically, microorganisms such as bacteria, yeasts, or moulds (starter culture) initiate the fermentation process either by their indigenous presence in the substrate or by being externally introduced. Among these, lactic acid bacteria (LAB) are the most common microorganisms utilised in food fermentation due to their vast adaptability to various environmental conditions and different origins of substrates (plant and animal), as well as their efficient proteolytic system [64,66]. During fermentation, proteolytic action of the starter culture converts protein-rich substrates into protein hydrolysate containing small bioactive peptides having different biological and functional properties [46].

Production of protein hydrolysate/peptides by microbial fermentation offers several advantages. For instance, the starter cultures used in fermentation have different characteristics and are capable of secreting diverse proteolytic enzymes that can efficiently break down proteins into hydrolysate mixtures possessing different characteristics [46,64]. In addition, microbial fermentation is a cost-effective approach as the starter cultures are relatively inexpensive. In addition, this technique is environmentally friendly as it does not produce any toxic substances, and the use of GRAS microorganisms produces much safer peptides, with fewer side effects [67]. Notably, the fermentation process can also degrade antinutritional factors present in the starting materials and enhance their flavour profile and overall acceptability [64,68]. On the other hand, microbial fermentation encounters a few disadvantages. The presence of other secondary metabolites, such as acids, in the medium can synergistically act with the produced hydrolysates to possess biological activities. However, the action of these metabolites can be eliminated by purification steps [66]. In addition, unlike enzymatic hydrolysis, microbial fermentation may face insufficient reproducibility from batch to batch when carried out on an industrial scale [46,67].

Numerous studies have investigated the fermentation process using different starter cultures to improve the digestibility, bioavailability, bioactivities, and sensory profile of microalgae [68,69,70,71,72,73,74,75,76,77,78,79]. However, fewer studies have focused on the production of bioactive peptides from microalgae through microbial fermentation. For instance, Yay et al. [13] hydrolysed *A. platensis* using co-culture fermentation with LAB (*Lactobacillus helveticus* B-4526) and yeast (*Kluyveromyces marxianus* Y-329). The resulting hydrolysates showed good antioxidant activity (DPPH radical scavenging) and antihypertensive (ACE inhibitory) effect without any cytotoxic effect on normal cell lines. In another study, antioxidant peptides were also generated through co-culture fermentation of *A. platensis* using the bacteria *Thermus thermophilus* HB27 and the yeast *Saccharomyces cerevisiae* CH006. The generated peptides exhibited in vitro DPPH and hydroxyl radical scavenging activity and effectively reduced ROS and melanin production in a zebrafish in vivo model [80]. Immunomodulatory peptides from *A. platensis* fermented using co-culture fermentation with *Lactobacillus plantarum* B7 and *Bacillus subtilis* 168 were successfully produced by An et al. [81]. The immunomodulatory effect of the produced peptides was investigated on murine primary splenic lymphocytes, where they promoted lymphocyte proliferation and modulated cytokine (IL-2 and IL-10) secretion.

Recently, studies have employed microbial fermentation combined with enzymatic hydrolysis (enzymatic-fermentation hydrolysis) for the production of bioactive peptides. Verni et al. [14] compared the effectiveness of fermentation (*Lactiplantibacillus plantarum* T0A10), enzymatic hydrolysis (Alcalase), and enzymatic-fermentation hydrolysis (Alcalase-*L. plantarum* T0A10) approaches on the generation of antioxidant and antibacterial peptides from *A. platensis*. They found that all treatments produced bioactive peptides with good antioxidant and antibacterial activities. However, peptides generated using alcalase alone had stronger antifungal activity against *Penicillium roqueforti*, while enzymatic-fermentation hydrolysis led to the production of peptides with more pronounced ABTS radical scavenging activity and higher antibacterial activity against *Escherichia coli*. Another research group [15] employed a similar approach to generate hypocholesterolemic peptides from *A. platensis*. In their study, they individually investigated enzymatic hydrolysis using peptidase R, fermentation using a mixture of starter cultures containing seven strains (*Lactobacillus casei*, *Lactobacillus acidophilus*, *Lactococcus latis subspecies lactise*, *Lactococcus latis subspecies cremoris*, *Lactococcus latis subspecies lavtis bv. diacetylactis*, *Saccharomyces cerevisiae*, and *Saccharomyces lactis*), and a combined approach involving fermentation followed by enzymatic hydrolysis (fermentation-enzymatic hydrolysis) to evaluate the hypocholesterolemic activity. They observed that the combined hydrolysis with fermentation and peptidase R yielded high soluble protein, peptide, and free amino acid content with strong hypocholesterolemic activity, as evidenced by potent inhibitory activity against 3-hydroxy-3-methylglutaryl-coenzyme A reductase.

Microbial fermentation, utilising bacteria and yeast as starter cultures, has been explored for the production of bioactive peptides from microalgae, demonstrating multiple health benefits such as antioxidant, antihypertensive, antibacterial, and immunomodulatory activities. Nevertheless, compared to enzymatic hydrolysis, studies on microbial fermentation remain limited, with existing research primarily focused on *A. platensis*. Therefore, more comprehensive investigations are needed to explore the potential of microbial fermentation in generating bioactive peptides from different species of microalgae.

### 2.3. Purification and Identification of Microalgae-Derived Peptides

Regardless of the production methods, generation of bioactive peptides typically results in crude protein hydrolysates containing a mixture of bioactive peptides and other impurities that may hinder their bioactivities. Consequently, purification of protein hydrolysates is necessary to obtain pure peptide fractions and to identify specific peptide sequences in the most active fractions. Although purification and identification are considered characterisation steps, they are integral to the overall workflow of peptide discovery, as they enable the link between peptide structure and function to be established. Generally, bioactive peptides can be purified, fractionated, and identified from the heterogenous mixture (protein hydrolysates) based on their physicochemical characteristics, such as molecular weight, polarity, or charge. The fractionation and purification of bioactive peptides commonly involve membrane filtration and chromatographic techniques, while peptide identification is based on analytical techniques such as mass spectrometry (Figure 1C).

Membrane separation technique is a physical process that separates molecules based on their molecular weight, hydrodynamic volume, and membrane pore size, driven by a pressure gradient [82]. Different types of membrane separation are typically classified according to membrane pore size, including microfiltration, ultrafiltration, nanofiltration, and reverse osmosis. Ultrafiltration, such as the use of molecular weight cut-off (MWCO) membranes, is the most widely applied technique for peptide fractionation and purification [67]. While ultrafiltration is a fast, cost-effective, and scalable process that can produce a high yield, it is considered a primary purification step because the fractionated product remains a mixture of peptides. Therefore, this technique is often combined with subsequent chromatographic purification steps to achieve higher purity. Chromatographic techniques fractionate peptides with high purity and involve interaction between peptides and the mobile and stationary phases. Size-exclusion chromatography/gel filtration chromatography (based on molecular size), ion-exchange chromatography (based on charge), and reversed-phase high-performance liquid chromatography (based on hydrophobic and hydrophilic properties) are the most applied chromatographic techniques to separate peptides from peptide mixtures with different properties [8,21,66,82].

After fractionation and purification, the most active fractions are further subjected to peptide sequencing and identification using mass spectrometric techniques. In this technique, peptides are ionised and separated based on their mass-to-charge ratio (*m*/*z*). The resulting ions are then detected and analysed to determine the peptides’ molecular weight and amino acid sequences. Electrospray ionisation (ESI) and matrix-assisted laser desorption/ionisation (MALDI) are the most common ionisation sources used in mass spectrometry for peptide identification. ESI is usually preceded by liquid chromatography (LC), which separates peptide mixtures based on hydrophobicity and other chemical properties before they enter the mass spectrometer. This combination, known as liquid chromatography-tandem mass spectrometry (LC-MS/MS), enhances sensitivity and resolution by reducing sample complexity prior to ionisation. ESI is typically coupled with a triple quadrupole (Q) or quadrupole-time-of-flight (Q-TOF) mass analyser, while MALDI is commonly paired with a time-of-flight (TOF) analyser. Therefore, the most popular techniques for peptide identification are ESI-LC-MS/MS and MALDI-TOF-MS/MS. Recently, hybrid mass spectrometers such as Q-TOF or TOF-TOF instruments have been developed to enhance mass accuracy and sensitivity. Additionally, the hybrid Q-TOF coupled with nano-ESI allows operation with lower buffer volume (low flow rate) and sample amounts [67,82]. The obtained mass spectrometric data are processed using various software, such as PEAKS studio and Mascot, which compare the observed spectra with existing databases to identify peptide sequences.

Peptide purification and identification is a complex process and can be influenced by the nature of the peptides present in the mixture. Therefore, a comprehensive multistep separation is often applied to achieve efficient peptide purification/identification with potent activity [19,20,56,59,83,84]. In a recent study conducted by Suo et al. [20], a series of chromatographic techniques were employed to separate, purify, and identify potent antihypertensive (ACE-inhibitory) peptides from trypsin-hydrolysed *C. pyrenoidosa*. The first purification step involved gel filtration chromatography with a Sephadex column, yielding five fractions, of which the third eluted fraction (CTF3) had the strongest ACE inhibitory activity of 54.86% at 400 μg/mL. Subsequent fractionation of CTF3 was conducted using ion-exchange chromatography with a Sepharose Fast Flow column, obtaining two fractions, CTD1 and CTD2, with CTD1 being the most effective in inhibiting ACE activity at about 60%. Finally, RP-HPLC using a C18 column was used and further increased the peptides activity against ACE to up to 80% (CTR1 and CTR2; the first eluted fractions). These fractions (the most active) were subjected to peptide identification using nano-LC-MS/MS with a Q Exactive Hybrid Quadrupole-Orbitrap MS and resulted in the identification of over 600 peptide sequences. These sequences were screened, based on score, mass error, ion intensity, and bioinformatic tools, into 10 potent ACE-inhibitory peptides with activity ranging from 17.93 to 97.60% at 400 μg/mL.

### 2.4. Bioinformatics and Computational Tools

Production of bioactive peptides through enzymatic hydrolysis primarily employs either single- or multi-protease systems. While this approach is effective, the process of selecting appropriate proteases for the preparation of bioactive peptides is often time-consuming and labour-intensive. As a result, researchers increasingly utilise computer-based (in silico) tools for the discovery of bioactive peptides (Figure 1D).

#### 2.4.1. In Silico Enzymatic Hydrolysis

This approach predicts the proteolysis of a protein substrate by specific enzymes with the aid of computer-based simulation and bioinformatics tools. It can predict the release of bioactive peptides from the parent protein (with a known amino acid sequence) and quickly screen multiple proteases, leading to reduced time and cost in selecting an appropriate protease prior to actual wet-lab experiments. In silico enzymatic hydrolysis involves the following three main components: (i) proteolysis simulation web servers, (ii) protein databases, and (iii) bioactive peptide databases.

The structures (amino acid sequences) of proteins of interest are retrieved from various protein databases such as the Research Collaboratory for Structural Bioinformatics Protein Data Bank (RCSB PDB) (https://www.rcsb.org/, accessed on 13 April 2025), the Universal Protein Resource (UniProt) (https://www.uniprot.org/, accessed on 13 April 2025), and the National Centre for Biotechnology Information (NCBI) (https://www.ncbi.nlm.nih.gov/protein/, accessed on 13 April 2025).

Several proteolysis simulation web servers are available, such as BIOPEP-UWM (https://biochemia.uwm.edu.pl/biopep-uwm/, accessed on 13 April 2025), which contains 34 different enzymes from microbes, vegetables, fruits, and humans; PeptideCutter (https://web.expasy.org/peptide_cutter/, accessed on 13 April 2025), which includes 34 different enzymes and chemicals; FeptideDB, which also contains 34 different enzymes; AHPP, which provides 10 enzymes from the gastrointestinal tract and 7 enzymes from vegetables and fruits; and SpirPep, which contains 10 enzymes.

To date, numerous bioactive peptide databases have been developed based on previously identified bioactive peptides from both in vitro and in vivo studies. These databases classify bioactive peptides based on various categories such as their source of origin and type of bioactivity. BIOPEP-UWM (https://biochemia.uwm.edu.pl/, accessed on 13 April 2025) is one of the most commonly used databases in peptide research, containing 5149 bioactive peptide sequences categorised into 58 different bioactivity groups. Other peptide databases such as DFBP (http://www.cqudfbp.net/, accessed on 13 April 2025), which contains 4652 peptide sequences classified into 31 bioactivity groups; AHTPDB (http://crdd.osdd.net/raghava/ahtpdb/, accessed on 13 April 2025), which reports only antihypertensive peptides with 1700 sequences; BioPepDB (http://bis.zju.edu.cn/biopepdbr/index.php, accessed on 13 April 2025), which includes 4807 bioactive peptides with antimicrobial, antihypertensive, and anticancer activities; BaAMPs (http://www.baamps.it/, accessed on 13 April 2025; currently offline) and YADAMP (http://yadamp.unisa.it/about.aspx, accessed on 13 April 2025; currently offline), which report only antimicrobial peptides.

A recent study reported by Villaró et al. [17] employed in silico enzymatic hydrolysis of *A. platensis* protein to screen different proteases for the generation of bioactive peptides with antioxidant, antihypertensive, and antidiabetic activities. The amino acid sequences of *A. platensis* proteins (phycocyanin and allophycocyanin) were obtained from the UniProt database and further in silico digested using pepsin, papain, thermolysin, and ficin through the PeptideCutter simulation web server. The generated peptide sequences were searched against BIOPEP-UWM to identify the number of bioactive peptides and compare their bioactivities. Their results showed that papain and ficin were the most efficient enzymes in releasing a larger number of bioactive peptides with ACE inhibitory peptides (366 and 300 peptides) DPP-IV inhibitory peptides (530 and 413 peptides), while ficin generated a larger number of antioxidant peptides (55 peptides). The multi-biological activities of protein hydrolysates generated with both enzymes (papain and ficin) were confirmed in vitro, displaying strong antihypertensive activity (ACE and renin), antioxidant activity (DPPH and FRAP), and antidiabetic activity (DPP-IV); however, they showed moderate antidiabetic activity against α-amylase and α-glucosidase. Another study virtually hydrolysed *A. platensis* protein C1 genome (PCC9438) retrieved from the NCBI database, using the web server SpirPep using a wide range of available proteases [85]. Among all the tested proteases, a larger number of bioactive peptides were obtained with proteinase K (1120 peptides), pepsin pH > 2 (1091 peptides), pepsin pH 1.3 (970 peptides), chymotrypsin (806 peptides), and thermolysin (729 peptides). The bioactive peptides were searched against the SpirPep database, with >90% of the peptide sequences possessing ACE inhibitory activity, and these findings were correlated with the in vitro proteolysis with the selected enzyme (thermolysin). Xie et al. [86] generated ACE inhibitory peptides through in silico gastrointestinal digestion (pepsin, trypsin, and chymotrypsin) of *C. vulgaris* proteins using the BIOPEP-UWM digestion tool. The identified ACE inhibitory peptides were effective in decreasing systolic blood pressure in vivo using spontaneously hypertensive rats.

#### 2.4.2. Quantitative Structure–Activity Relationship (QSAR)

Production of bioactive peptides by any hydrolysis method (conventional or in silico) leads to the identification of a large number of peptides, many of which are not all necessarily bioactive. Therefore, identifying bioactive peptides from protein hydrolysates remains a major challenge. In recent years, the QSAR technique has been introduced for the discovery of food bioactive peptides. QSAR is an in silico approach employed to screen and identify bioactive peptides from a peptide pool based on the relationship between their structural/physicochemical properties and their specific bioactivity [87]. This approach involves multiple and series steps, which includes (i) data collection from literature or peptide databases to build a peptide library with known activity; (ii) peptide classification based on specific molecular descriptor (1D to 4D levels) of their different structural and physicochemical features such as size, charge, hydrophobicity, and electronic properties; (iii) model training and validation using mathematical equations and computational models such as principal component analysis (PCA), multiple linear regression (MLR), partial least square regression (PLSR), and artificial neural network (ANN); and finally (iv) prediction and screening: peptides with the highest predicted bioactivity are synthesised and validated in wet-lab testing [88].

To date, QSAR studies on microalgae-derived bioactive peptides have been performed to identify antioxidant peptides. A QSAR model was employed to screen and identify effective antioxidant peptides derived from protein hydrolysates of a co-cultured *C. pyrenoidosa* and *Yarrowia lipolytica* [87]. The dataset containing 25 antioxidant peptides (based on oxygen radical absorbance capacity) obtained from the published literature was randomly divided into a calibration set and a prediction set. The model incorporated electronic descriptors related to amino acid side chains, including charge and hydrophobicity, and was analysed using PLS regression. The model demonstrated high predictive performance with cumulative multiple correlation coefficient (R^2^) and cumulative cross-validation coefficient (Q^2^) of 0.999 and 0.587, respectively, for the calibration set. The model successfully predicted three antioxidant peptides, including AGYSPIGFVR with the highest predicted antioxidant activity, followed by VLDELTLAR and LFDPVYLFDQG. Subsequent confirmatory testing using HepG2 cells confirmed the model’s prediction, whereby the antioxidant capacity of the three peptides followed the same order predicted by the QSAR model. In another study, Zhu et al. [89] applied QSAR modelling to identify antioxidant peptides from *A. pyrenoidosa* protein hydrolysates based on DPPH radical scavenging activity. A total of 69 antioxidant peptides with DPPH radical scavenging activity were collected from previously published studies. Four QSAR models were constructed with two types of amino acids descriptors, including the vector of radial distribution descriptors and geometrical descriptors (SVRG) and the vector of principal component scores for electronic eigenvalue descriptors (SVEEVA). The employed models achieved good predictive performance, with training set R^2^ between 0.856 and 0.921 and prediction R^2^ ranging between 0.507 and 0.714. Five antioxidant peptide candidates were selected out of the 25 peptides used in the QSAR models and synthesised for further activity confirmation. Among the five peptides, AGWACLVG was the most active candidate with a DPPH radical scavenging (IC_50_ of 68.88 µM) and ABTS radical scavenging activity (6.90 mM Trolox equivalent/g), which were superior to glutathione (DPPH IC_50_ = 520.62 µM), while YPLDL and IDLAY also demonstrated potent ABTS radical scavenging activity of 6.77 and 6.20 mM Trolox equivalent/g. Despite an approximate 60% prediction accuracy due to limitations in dataset size and assay variability compared to those sequences used in the training set, the study demonstrated the utility of QSAR models as a screening tool and emphasised the need for expanded databases to improve model robustness and accuracy.

#### 2.4.3. Molecular Docking

Molecular docking is a powerful in silico technique widely used in the discovery of bioactive molecules (e.g., peptides) and is often applied in conjunction with QSAR modelling. The molecular docking process involves simulating the interaction between a ligand (e.g., bioactive peptides) and a receptor (e.g., target enzymes/proteins). This technique provides insights into the molecular basis of ligand-receptor interactions and the preferential orientation of the ligand within the receptor’s binding pocket, scoring the interactions based on the bonding types (hydrogen bonds, hydrophobic interactions, π-π stacking, π-cation, and/or Van der Waals forces) and binding free energy (ΔG) [88]. A stronger ligand-receptor interaction is characterised by a more negative binding free energy (kcal/mol) [90]. Peptide discovery using computational tools such as molecular docking is a rapid and cost-effective approach that can screen a large number of peptides for a specific bioactivity. However, confirmatory studies are essential to confirm the activity of the selected bioactive peptides.

Several software packages, such as AutoDock 4 (https://autodock.scripps.edu/download-autodock4/, accessed on 1 May 2025), AutoDock Vina (http://vina.scripps.edu/, accessed on 1 May 2025), AutoDockFR (https://ccsb.scripps.edu/adfr/, accessed on 1 May 2025), and AutoDock CrankPep (https://ccsb.scripps.edu/adcp/documentation/, accessed on 1 May 2025), are freely available for academic use to perform molecular docking studies, while some software, such as Gold (https://www.ccdc.cam.ac.uk/solutions/csd-discovery/components/gold/, accessed on 1 May 2025), Glide (https://www.schrodinger.com/products/glide, accessed on 1 May 2025), and CDOCKER (https://discover.3ds.com/discovery-studio-visualizer-download, accessed on 1 May 2025), require a purchased licence.

A number of studies have been reported on microalgae-derived peptides discovered with the aid of molecular docking. For example, molecular docking has been employed to identify potent ACE inhibitory peptides from different protein hydrolysates, including *C. vulgaris* [86], *A. platensis* [83], and *C. pyrenoidosa* [20]. These studies performed molecular docking between the identified peptides from each hydrolysate and the ACE crystal structure of human testis enzyme (PDB ID: 1O86) and selected the top peptides with strong binding affinities for further synthesis and activity confirmation. This approach enabled the discovery of novel and strong ACE inhibitory peptides with low IC_50_: two tripeptides, TTW (IC_50_ = 0.61 µM) and VHW (IC_50_ = 0.91 µM) from *C. vulgaris* [86]; a hexapeptide, TVLYEH (IC_50_ = 2.88 µM), and a heptapeptide, LQAGGLF (IC_50_ = 66.83 µM), from *A. platensis* [83]; and three pentapeptides, LKKAP (IC_50_ = 36.19 µM), LVAKA (IC_50_ = 26.66 µM), and PGLRP (IC_50_ = 44.78 µM), from *C. pyrenoidosa* [20]. In addition, antidiabetic peptides derived from *A. platensis* have been successfully obtained using bioinformatics tools, including peptide ranker for general activity prediction, followed by molecular docking of the identified peptides with α-amylase (PDB ID: 1BAG), α-glucosidase (3WY1), and DPP-IV (2AJB). The top three peptides, namely GVPMPNK, RNPFVFAPTLLTVAAR, and LRSELAAWSR, were further synthesised and exhibited strong inhibitory activity against α-amylase (IC_50_ = 264.20–608.13 μM), α-glucosidase (IC_50_ = 92.83–204.45 μM), and DPP-IV (IC_50_ = 102.26–259.51 μM) [91]. A recent study performed a series of bioinformatics analysis on *A. platensis* protein (allophycocyanin) to discover anti-ageing peptides (anti-tyrosinase inhibitory activity) through in silico enzymatic hydrolysis with pepsin, trypsin, and chymotrypsin, followed by refining the generated peptides based on their predicted solubility and toxicity (https://www.innovagen.com/proteomicstools, accessed on 1 May 2025), and finally molecular docking between the top ten peptides and tyrosinase (PDB ID: 2Y9X) was performed using CDOCKER software (https://discover.3ds.com/discovery-studio-visualizer-download, accessed on 1 May 2025). The process enabled the discovery of the tripeptide DER, which showed strong tyrosinase inhibitory activity at a low IC_50_ of 1.04 mM, which was superior to the known tyrosinase inhibitor arbutin with an IC_50_ of 5.73 mM [92].

## 3. Health Benefits, Mechanism and Structure–Activity Relationship of Microalgae-Derived Bioactive Peptides

### 3.1. Antioxidant Peptides

Oxidative stress is a state of imbalance between the production of reactive oxygen species (ROS) and the cellular antioxidant defence system [49]. ROS are highly reactive molecules that are produced within the body for regulating cell homeostasis, including viability, proliferation, migration, and differentiation [19,93]. Oxidative/redox balance is naturally maintained by the cellular defence mechanism through biochemical pathways that activate antioxidative enzymes [94,95]. However, when there is overproduction of reactive molecules, it can overwhelm the defence system of cells, leading to damage of the cellular components such as DNA, proteins, and lipids, leading to functional disruption and subsequent cell death [19,26,96]. Moreover, oxidative stress has been linked to the initiation and progression of a variety of chronic diseases such as cardiovascular disease, diabetes, neurodegeneration, and cancer [26,29,97,98]. Therefore, antioxidant agents are a key factor in supporting human antioxidant defences and effectively eliminating and detoxifying the negative effects of oxidative species. In this context, natural antioxidant peptides derived from microalgae have gained significant attention due to their ability to scavenge free radicals, chelate metal ions, inhibit lipid peroxidation, and regulate endogenous antioxidative enzymes, offering a promising alternative to synthetic antioxidant compounds with potential application in the pharmaceutical and food industries.

Antioxidant peptides have been successfully produced from different species of microalgae, including *Arthrospira platensis* [48,49,93,95,96,99,100,101,102,103,104], *Chlorella pyrenoidesa* [87], *Auxenochlorella pyrenoidosa* [89], *Haematococcus pluvialis* [105,106], *Isochrysis Zhanjiangensis* [59,94,107,108], *Tetradesmus obliquus* [109], *Schizochytrium limacinum* [110], and *Synechococcus* sp. VDW [111] which has exhibited potent antioxidant activity (Table S1). The cell-free antioxidant activity of microalgae-derived antioxidant peptides demonstrated significant antioxidant effects via free radical scavenging and metal-chelating activity. For instance, the cell-free antioxidant activity of the underutilised microalgae *H. pluvialis* was recently reported by Zhang et al. [105], who isolated three peptides, GVFHPGPF (IC_50_ = 0.33–1.10 mg/mL), IDPGTWRPL (IC_50_ = 0.14–0.52 mg/mL), and APIIGKPAPGFK (IC_50_ = 0.25–0.47 mg/mL), that demonstrated strong DPPH, ABTS, and hydroxyl radical scavenging activity. Meanwhile, two peptides, namely EYFDALA and GMCCSR, derived from *A. platensis* possessed excellent DPPH (IC_50_ = 5.80 and 36.93 μM) and ABTS (IC_50_ = 13.40) and (16.94 μM) radical scavenging activity in a dose-dependent pattern, showing comparable radical scavenging activity to the positive control vitamin C (IC_50_ = 27.42 μM and 26.18 μM) [96,102].

**Figure 2:**
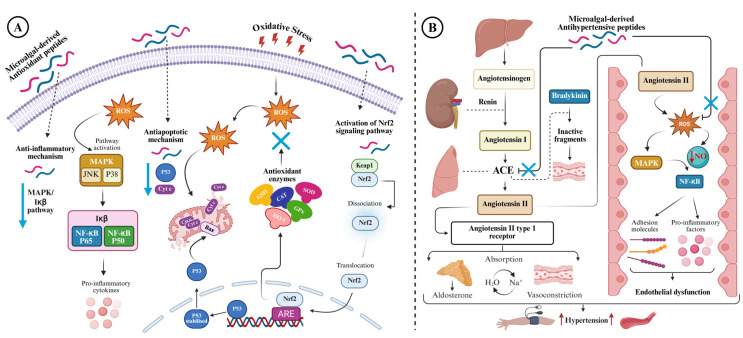
Action mechanism of microalgae-derived peptides in mitigating oxidative stress, inflammation, and hypertension: (**A**) Microalgae-derived antioxidant peptides inhibit oxidative stress by scavenging ROS and activating the Nrf2 signalling pathway, promoting the expression of antioxidant enzymes. These peptides also protect against cellular inflammation and apoptosis by suppressing the NF-κB/MAPK signalling pathway and stabilising p53 and inhibiting cytochrome c release. (**B**) Microalgae-derived antihypertensive peptides interfere with the RAAS by inhibiting ACE activity and reducing Ang II production. In addition, the antihypertensive peptides suppress ROS formation and downregulate NF-κB/MAPK-mediated endothelial dysfunction, reducing pro-inflammatory factors and adhesion molecules, ultimately alleviating hypertension. Arrows indicate activation or direction of pathways; blue downward arrows show downregulation due to peptide action, and blue “X” indicates inhibition by peptide action. Created in BioRender. Qoms, M. (2025) https://BioRender.com/nvuth2e, accessed on 21 August 2025.

The action mechanism of microalgae-derived antioxidant peptides in alleviating oxidative stress in cells is a complex process, involving multiple molecular reactions and biochemical pathways. Several research groups have investigated the cellular antioxidant capacity of microalgae-derived peptides to gain insights into their mode of action in mitigating cellular oxidative stress and modulating antioxidant enzymes and gene expression. *A. platensis* peptides alleviated hydrogen peroxide (H_2_O_2_)-induced oxidative stress in human immortal keratinocyte (HaCaT) cells (INSSDVQGKY) [49] and human leucocytes (NPLSTQDDVAASL, LGLDVWEHAYYL, and GGTCVIRGCVPKKLM) [99,100,101]. These peptides scavenged the intracellular ROS generated by H_2_O_2_ in a concentration-dependent manner without any toxic effect on the tested cells. Microalgae-derived peptides trigger the activation of the Nrf2 (nuclear factor erythroid 2-related factor 2) pathway, a crucial mechanism in mitigating oxidative stress, whereby Nrf2 dissociates from its inhibitor Keap1 (Kelch-like ECH-associated protein 1) and translocates into the nucleus, promoting the expression of antioxidant enzymes such as superoxide dismutase (SOD), catalase (CAT), glutathione reductase (GR), heme oxygenase-1 (HO-1), and glutathione peroxidase (GPx). For instance, the antioxidant peptides derived from *I. zhanjiangensis* restored the viability of H_2_O_2_-stressed human neuroblastoma (SH-SY5Y) cells (peptides IIAVEAGC, IIAVE, and AGC) [94] and H_2_O_2_-stressed SH-SY5Y or human umbilical vein endothelial (HUVEC) cells (peptide AYAPE) [107] via the activation of the Nrf2/Keap1 signalling pathway and upregulated the expression of antioxidant enzymes such as HO-1, SOD, and GPx. Furthermore, these peptides protected cellular inflammation and apoptosis by suppressing phosphorylation of nuclear factor kappa-light-chain-enhancer of activated B cells/mitogen-activated protein kinase (NF-κB/MAPK) signalling pathway, downregulating the expression of p53, and inhibiting cytochrome c release. These findings suggest that microalgae-derived antioxidant peptides can function as anti-inflammatory/anti-apoptotic agents, making them a promising candidate for mitigating oxidative stress-related inflammation (Figure 2A).

In vivo studies on the antioxidant activity of peptides derived from *A. platensis* have been investigated using H_2_O_2_-induced oxidative stress in zebrafish larvae models [93,95,103,104], demonstrating promising therapeutic potential for oxidative stress-related diseases. The *A. platensis*-derived peptide GGGAFSGKDPTKVDR reduced the intracellular ROS level and prevented stress-mediated apoptosis in a dose-dependent pattern (5–45 μM) by upregulating antioxidant enzymes (SOD and CAT) and the expression of mRNA levels of antioxidant enzyme genes (GPx, GST, GCS), while reducing malondialdehyde (MDA, lipid peroxidation). Interestingly, this peptide was safe for zebrafish larvae and maintained normal hatching rate, mortality rate, heart rate, and morphology [95]. Additionally, microalgae-derived peptides showed in vivo antioxidant effects when investigated in the *Caenorhabditis elegans* (nematode) model. Liu et al. [49] observed that the *A. platensis* peptide fraction (F2) effectively increased the survival rate of nematodes at 0.2 mg/mL by 35%, 50%, and 24% after exposure to H_2_O_2_, paraquat (pesticide), and heat stress at 35 °C, respectively. The antioxidant effect was confirmed by the ability of the peptide fraction to scavenge intracellular ROS and upregulate antioxidative enzymes SOD and GPx while decreasing the MDA content. Similarly, the *H. pluvialis*-derived peptide KFTPAP at 100 μM enhanced the activity of endogenous antioxidant enzymes (SOD and CAT) and reduced the MDA content in nematodes [106].

Structural features of antioxidant peptides such as molecular weight (MW), peptide chain length, amino acid sequence, and composition are associated with their biological function via structure–activity relationship. As shown in Table S1, a total of 35 microalgae-derived antioxidant peptide sequences with known effective concentrations were identified. These peptides consisted of short chains ranging between 3 and 16 amino acid residues with low MW of <2 kDa (249–1708 Da). Low-MW peptides possess potent biological activity and high bioavailability because they are easily absorbed and can penetrate the cell membrane, subsequently mitigating cellular oxidative stress. A recent study by Zhang et al. [105] aimed to produce antioxidant peptides from the microalga *H. pluvialis* and investigate the effect of MW on their activity. The results showed that the lower MW peptide fraction (<5 kDa) had better DPPH radical scavenging activity compared to the higher MW fraction (>5 kDa). This low MW fraction possessed strong antioxidant activity in D-galactose-induced liver oxidative stress in ageing mice at 200 mg/kg body weight/day (equivalent to 1.2 g/day for humans) through the activation of the Nrf2/Keap1 signalling pathway and increased the activity of liver antioxidant enzymes (SOD, GPx, and HO-1), with a simultaneous decrease in MDA and hepatic chronic inflammation-related cytokines TNF-α and IL-6. Moreover, the peptide treatment modulated the intestinal gut microbiota and their secondary metabolites by increasing short-chain fatty acids (SCFAs) and branched-chain fatty acids (BCFAs), while decreasing the content of lipopolysaccharides (LPS) in the D-galactose-induced mice, thereby modulating liver function as the peptides crossed the intestinal barrier and reached the liver through blood circulation. Antioxidant peptides generated from *C. vulgaris* protein using pepsin and promod [26] and alcalase [19] were separated into >10 kDa, 3–10 kDa, and <3 kDa, wherein the lowest MW peptide fractions exhibited stronger radical scavenging activity.

Another crucial structural feature of bioactive peptides is the amino acid composition and their positions. Analysis of 35 microalgae-derived antioxidant peptides (Table S1) revealed that all the sequences contained hydrophobic amino acids, with approximately 77% of the sequences containing more than 50% hydrophobic residues, such as Leu, Ile, Ala, Pro, Val, Phe, Trp, Gly, and Met. The presence of hydrophobic amino acid residues in peptides facilitates their diffusion into the cell membrane lipid bilayer through hydrophobic interactions and enables them to transfer chelators by donating hydrogen, metal ions, or scavengers of free radicals, thereby contributing to their free radical scavenging activity and preventing lipid peroxidation [48,87,104,112]. Moreover, aromatic amino acids including Phe, Trp, and Tyr were found in 63% (22 peptide sequences); these residues have been associated with potent free radical scavenging activity via direct electron transfer [87,89,109]. Similarly, basic amino acids, including Lys, Arg, and His, were found in 66% of the microalgae-derived antioxidant peptides. The imidazole ring-containing His strongly contributes to the metal chelating and radical scavenging activity of antioxidant peptides by the hydrogen atom/single electron transfer capacity of its imidazole ring [48,113]. While sulphur-containing amino acids Met and Cys were found in 10 peptide sequences, which represent about 29% of the total peptides, they can contribute to antioxidant activity by direct radical scavenging [101]. The position of specific amino acid residues within peptide sequences, particularly at both ends (N- and C-terminal), has a crucial effect on their biological activity. As shown in Table S1, the N- and/or C-terminus of microalgae-derived antioxidant peptides were predominantly occupied by hydrophobic amino acids, particularly the residues Ala, Ile, Gly, and Val located at the N-terminal. Another observation is that basic amino acids, mainly Arg, followed by Lys and His, were found in most of the sequences at either end of the peptides (14 out of 35 peptides), especially Lys at the C-terminal. Meanwhile, a few of the N-terminal and/or C-terminal microalgae peptides were composed of acidic amino acids Glu and Asp (four peptides) and the sulphur-containing amino acids Cys and Met (three peptides). These residues’ patterns at the antioxidant peptides termini have also been supported by structure–activity relationship studies such as molecular docking. Lin et al. [94] studied the molecular interaction of the antioxidant peptide IIAVEAGC, derived from *I. zhanjiangensis*, and its truncated sequences IIAVE and AGC with Keap1 using molecular docking. The results demonstrated that all the peptides interacted with Keap1 through hydrogen bonds, and the key interacting amino acid residues were Ile at the N-terminal of the octa- and penta-peptide, while Cys, at the C-terminus of the tripeptide. Additionally, Ala at the N-terminal of the peptide AYAPE, with the contribution from Tyr, Pro, and Glu, played a critical role in interacting with Keap1 and Bax (Bcl-2-associated X protein; a pro-apoptotic protein) through hydrogen bonds [107].

In addition to the molecular weight, chain length, amino acid composition, and residue position, the secondary structure of peptides has also been shown to influence their antioxidant potential. The secondary structure determines conformational stability and the extent to which bioactive residues are exposed, thereby modulating radical scavenging activity and protective effects in biological systems. Although studies on the relationship between the secondary structure of microalgae-derived antioxidant peptides and their activities are limited, a few recent reports have investigated their secondary structure composition using circular dichroism (CD). Wang et al. [19] demonstrated that peptide fractions from *C. vulgaris* with molecular weight < 3 kDa were dominated by β-sheet (47.00%), followed by random coil (27.30%), β-turn (19.70%), and α-helix (13.70%), whereas larger peptides (>3 kDa) with lower activity were dominated by random coil (38.30%), followed by β-sheet (34.30%), β-turn (18.70%), and α-helix (17.50%). Simulated gastrointestinal digestion of these peptide fractions reduced α-helix and increased β-sheet structures, reflecting peptide unfolding and aggregation. These conformational changes enhanced their antioxidant activity during gastric digestion by exposing active residues, while intestinal digestion of the lower peptide fraction led to an increase in α-helical and a decrease in β-sheet structures, which negatively influenced antioxidant activity. Similarly, Zeng et al. [96] found that two peptides (EYFDALA and VTAPAASVAL) discovered from *A. platensis* exhibited superior antioxidant activity that was largely attributed to their high proportion of β-sheet structure (53.80 and 58.60%) and low proportion of α-helical content (8.30 and 9.60%). The highly ordered structure (>50%) was proposed to maintain conformational stability and promote interactions that enhanced radical scavenging and protection against erythrocytes haemolysis. In agreement, the most active antioxidant fraction obtained from fermented *A. platensis* was also composed predominantly of β-sheet (36.30%) with low α-helical content (11.40%) [80]. Collectively, these findings suggest that β-sheet enrichment represents a favourable structural feature contributing to antioxidant activity, whereas high α-helical content is less advantageous. Thus, secondary structure should be considered alongside other primary structural determinants when evaluating the structure–activity relationship of microalgae-derived peptides.

### 3.2. Antihypertensive Peptides

Hypertension, also known as high blood pressure, is often referred to as the silent killer because it does not have noticeable symptoms. Globally, hypertension affects about 1.3 billion adults aged between 30 and 79, and it is a major risk factor for cardiovascular disease (CVD), causing damage to blood vessels, kidneys, and the heart [20,114]. The clinical presentation of hypertension is indicated when systolic blood pressure and diastolic blood pressure exceed 140 and 90 mmHg, respectively [115]. The renin–angiotensin–aldosterone system (RAAS), a complex system of hormones and enzymes, is one of the key metabolic mechanisms involved in regulating blood pressure by balancing sodium and water absorption and vascular tone [116]. In this process, angiotensinogen, synthesised in the liver, is cleaved by the renin enzyme produced by the kidney to form angiotensin I (Ang I) [116]. Then, ACE enzyme, a dipeptidyl carboxypeptidase that belongs to the zinc metalloenzymes, converts Ang I into Angiotensin II (Ang II), which subsequently binds to the angiotensin II type 1 receptor (ATR1) in the ACE-Ang II-ATR1 axis, causing various physiological effects including vasoconstriction, secretion of aldosterone (ALD), increased absorption of water and sodium ions, and elevated blood pressure [117,118]. Another axis involved in the regulation of RAAS is the ACE2-Ang-(1-7)-Mas axis. In this axis, ACE2 cleaves Ang II and releases Ang-(1-7), which binds to the G protein-coupled receptor Mas and stimulates nitric oxide (NO) release from endothelial cells, exerting antihypertensive effects through vasodilation and antiproliferation, unlike the ACE-Ang II-ATR1 axis, which promotes vasoconstriction [119,120]. On the other hand, ACE can degrade the hypotensive peptide bradykinin, a part of the kallikrein-kinin system (KKS), thereby increasing blood pressure [22,121]. Therefore, modulating ACE activity is an important strategy in the management of hypertension. To date, several antihypertensive drugs based on ACE inhibitory activity have been developed and are commercially available, such as ‘pril’ medications like captopril, lisinopril, enalapril, and perindopril. However, these drugs are associated with numerous adverse health complications such as cough, edoema, skin rashes, taste alterations, and hyperkalaemia [22,43,122]. For this reason, natural bioactive peptides are being increasingly explored as alternative therapies for hypertension due to their minimal side effects. Figure 2B displays the general mechanism by which microalgae-derived antihypertensive peptides exert their effects in alleviating hypertension.

As shown in Table S2, different microalgae species, including *Arthrospira* sp. [83,123], *A. platensis* [48,85,114,119,120,124,125], *C. pyrenoidosa* [20], *C. sorokiniana* [51], *C. vulgaris* [86], *I. galbana* [126], *I. zhanjiangensis* [122,127], *N. oculata* [128], and *T. obliquus* [109] have been reported as good precursors for the generation of ACE-inhibitory peptides. These peptides demonstrated potent in vitro ACE inhibitory activity at low concentrations, with some peptides having an IC_50_ at the nanomolar level. Suo et al. [20] employed a combination of in vitro and in silico approaches to discover novel and potent ACE-inhibitory peptides from *C. pyrenoidosa*. Three pentapeptides, including LKKAP, LVAKA, and PGLRP, possessed strong in vitro ACE inhibitory activity with an IC_50_ of 36.19, 26.66, and 44.78 μM, respectively. These peptides also maintained their ACE inhibitory activity under a wide range of pH values (2 to 12) and temperatures (20–100 °C). Additionally, they maintained high stability under simulated gastrointestinal conditions by releasing new di- and tripeptides AP, RP, VAK, and PGL with low IC_50_ of 230.00, 15.20, 85.43, and 13.93 μM, respectively. Another study enzymatically hydrolysed *C. sorokiniana* protein, generating four novel ACE-inhibitory dipeptides, VW, IW, LW, and WV, exhibiting strong activity with low IC_50_ of <308 μM, with the first two sequences being the most potent ones, having a nanomolar IC_50_ of 0.58 μM (580 nM) and 0.50 μM (500 nM) [51]. Numerous studies reported the ACE-inhibitory activity of peptides derived from *A. platensis*. For instance, a recent study by Safitri and Hsu [124] discovered a novel pentapeptide ILLYR with a low IC_50_ of 10.54 μM, which was relatively stable under simulated gastrointestinal conditions with a 2-fold increment in its activity (IC_50_ = 23.35 μM). The mode of action of microalgae ACE-inhibitory peptides was studied using enzyme kinetics in the absence and presence of different concentrations of the peptides, whereby the Lineweaver-Burk plot is used to represent the data by transforming the nonlinear Michaelis-Menten equation into a linear relationship (reciprocal of reaction velocity (1/V) against the reciprocal of substrate concentration (1/[S])), thereby enabling the determination of kinetic parameters: the Michaelis-Menten constant (Km) (slope) and the maximum reaction velocity (Vmax) (y-intercept) [129]. The inhibition mechanism of all the investigated microalgae-derived ACE-inhibitory peptides was characterised by a non-competitive mechanism, except the peptides TVLYEH and LQAGGLF, which competitively inhibited the ACE. The non-competitive mechanism indicates that the Vmax is reduced with increasing the peptide concentrations, thereby reducing the maximum reaction rate, while Km remains unaffected with increasing the peptide concentrations, indicating that the peptides do not directly affect the binding affinity between the ACE and its substrate. Subsequently, the peptides interact with the ACE at an allosteric site, causing conformational changes to the ACE structure, thus altering its catalytic efficiency. Conversely, the competitive inhibition mechanism is characterised by an increased Km value without affecting the Vmax with increasing the peptide concentrations, indicating that the peptides compete with the substrate for binding to the active site of ACE, consequently reducing the amount of free enzyme available to bind to the substrate.

Endothelial dysfunction is a major contributor to the progression and pathogenesis of hypertension [22]. Ang II, a potent vasoconstrictor, can induce vascular dysfunction-related factors such as ROS and endothelin-1 (ET-1), which reduce the bioavailability of endothelial NO, a vasodilator agent, and activate nuclear factor κB (NF-κB), contributing to vascular inflammation. Also, these factors can induce cellular oxidative stress and inflammation, thereby leading to endothelial injury and apoptosis [22,122,127,130]. Thus, improving endothelial dysfunction and restoring endothelial NO bioavailability to prevent oxidative stress and vascular inflammation is considered a promising strategy for managing hypertension. In this context, the antihypertensive potential of microalgae-derived ACE-inhibitory peptides was investigated in an Ang II-induced human umbilical vein endothelial cell (HUVEC) model [122,123,127]. For example, the ACE-inhibitory peptide EMFGTSSET derived from *I. zhanjiangensis* with a low IC_50_ of 15.08 μM exhibited an antihypertensive effect via anti-inflammatory and anti-apoptosis mechanisms in Ang II-induced HUVEC [122]. The cells were pretreated with EMFGTSSET at 10, 50, and 100 μM for 1 h, followed by treatment with 10 μM Ang II to induce oxidative stress and inflammatory response (endothelial dysfunction). In a dose-dependent effect, the peptide (EMFGTSSET) treatment reduced the production of ROS, pro-inflammatory cytokines (IL-8, IL-1β, and TNF-α), pro-inflammatory enzymes (iNOS and COX-2), and ET-1. Additionally, peptide treatment downregulated the expression of ATR1 and adhesion molecules, including intracellular adhesion molecule 1 (ICAM-1) and vascular cell adhesion molecule 1 (VCAM-1). Ang II activates the MAPK signalling pathway (including p-p38, p-ERK, and p-JNK), which contributes to the activation of NF-κB and subsequent inflammatory responses. The peptide downregulated the expression of NF-κB by inhibiting the phosphorylation of MAPK components, thereby suppressing the inflammatory signalling cascade. Moreover, this peptide inhibited Ang II-induced apoptosis and alleviated endothelial injury by increasing the expression of proliferation-related protein Bcl-2 and downregulating the expression of p-Akt, caspase-3, caspase-9, and Bax.

The antihypertensive effect of microalgae-derived peptides was confirmed in vivo using spontaneously hypertensive rats (SHR), demonstrating potent and long-lasting effects with a promising potential to substitute synthetic commercial drugs like Captopril and Lisinopril. The ACE-inhibitory peptide EMFGTSSET was orally administered to SHR at 10 mg/kg body weight, inducing a significant reduction in systolic blood pressure (≈10 mmHg), which was maintained for 10 h, similar to Captopril at the same concentration [122]. Xie et al. [86] discovered two nanomolar ACE-inhibitory tripeptides (TTW; IC_50_ = 610 nM and VHW; IC_50_ = 910 nM) from *C. vulgaris*, and both peptides were orally administered to spontaneously hypertensive rats at a low concentration of 5 mg/kg body weight, reducing blood pressure. The peptide VHW significantly reduced systolic blood pressure within 2 h of oral administration, with the greatest drop from 234 to 184 mmHg, with a longer effect (−31 mmHg) than Lisinopril (−10 mmHg) after 8 h. Meanwhile, the other peptide, TTW, significantly decreased diastolic blood pressure from 180 to 140 mmHg within 2 h, compared to VHW (174 to 153 mmHg). Therefore, the two peptides can be used to treat different hypertensive phenotypes, or they may synergistically cooperate due to their distinct effects on systolic and diastolic blood pressure. Pan et al. [120] and Zheng et al. [119] investigated the in vivo mechanism of the antihypertensive effect of the *A. platensis*-derived tripeptides IQP (IC_50_ = 5.77 μM) and VEP (IC_50_ = 27.36 μM) after long-term oral administration (6 weeks) at 10 mg/kg body weight/day to spontaneously hypertensive rats. These peptides significantly decreased systolic blood pressure, diastolic blood pressure, left ventricular mass index, and right ventricular mass index. Moreover, they regulated the component of the ACE-Ang II-ATR1 axis (downregulated the expression of ACE, Ang II, and ATR1) and the ACE2-Ang-(1-7)-Mas axis (upregulated the expression of ATR2, ACE2, Ang-(1-7), and Mas receptor). Recent evidence has highlighted alternative pathways involving endothelial nitric oxide signalling as critical mediators of vascular homeostasis and blood pressure regulation. Carrizzo et al. [125] observed that *A. platensis*-derived peptide GIVAGDVTPI significantly lowered systolic blood pressure by ≈43 mmHg following a single oral administration of 10 mg/kg in SHR. The peptide also exhibited a dose-dependent vasorelaxation in isolated mesenteric arteries, an effect abolished by pretreatment with the nitric oxide synthase (NOS) inhibitor L-NAME and absent in vessels from endothelial nitric oxide synthase (eNOS)-knockout mice, indicating that its vasodilatory effect is NO dependent. Furthermore, pharmacological inhibition of PI3K and AKT suppressed the vasorelaxant response to the peptide GIVAGDVTPI, whereas inhibition of AMPK did not, suggesting that the peptide activated the PI3K/AKT/eNOS signalling pathways and increased endothelial NO production. Additionally, the peptide improved acetylcholine-induced vasorelaxation and elevated serum nitrite levels, supporting an NO-mediated mechanism underlying its antihypertensive effect.

To gain insights into the structural features and structure–activity relationships of microalgae-derived antihypertensive peptides, we critically analysed the peptides reported in the past 10 years (only those with confirmed activity in cell-free assay or cell/in vivo model). As displayed in Table S2, a total of 34 antihypertensive peptide sequences have been discovered and identified from different species of microalgae. These peptides exhibited strong cell-free ACE-inhibitory activity with IC_50_ values ranging between 0.35 μM (LRAKA) and 1971.50 μM (PTGNPLSP) and were effective in vivo in a hypertensive rat model with a dose ranging between 5 and 20 mg/kg body weight. The microalgae-derived antihypertensive peptides possessed short chain lengths varying from 2 to 14 amino acid residues, with tripeptides and pentapeptides each accounting for 15% (5 peptides), followed by dipeptides, heptapeptides, and decapeptides, each representing 12% (4 peptides). These peptides featured low molecular weights ranging between 303 and 1325 Da. Thus, they can be characterised by short sequences (2–14 residues) and low molecular weight (303–1325 Da). These characteristics enable them to efficiently interact with their target enzymes and enhance their bioavailability in the body. Investigations on the effect of MW on the antihypertensive activity of microalgae-derived peptides have been reported by several research groups. For instance, Lin et al. [51] found that peptide fractions from *C. sorokiniana* with lower MW of 270–340 Da exhibited stronger ACE inhibitory activity (IC_50_ = 0.015 mg/mL) compared to fractions with higher MW between 370 and 1400 Da (IC_50_ = 0.016–0.045 mg/mL). Similarly, lower MW peptides (<5 kDa) obtained from gastrointestinally hydrolysed Spirulina sp. protein possessed stronger ACE inhibitory activity (IC_50_ = 0.66 mg/mL) than higher MW fractions (5–100 kDa), with IC_50_ varying between 0.96 and 1.06 mg/mL [123]. Pekkoh et al. [18] also observed that the lower MW peptide fraction of <3 kDa (IC_50_ = 2.95 µg/mL) derived from *Chlorella* spp. had an 8-fold higher ACE inhibitory activity than the >3 kDa fraction (IC_50_ = 23.22 µg/mL). All the microalgae ACE-inhibitory peptides contained at least 1 hydrophobic amino acid residue (Leu, Ile, Ala, Pro, Val, Phe, Trp, Gly, and Met), with about 77% of the peptide sequences (26 peptides) containing >50% hydrophobic residues. Additionally, the presence of specific amino acid residues at either end of the peptides (N/C-terminal) is crucial for their activity. In this respect, we found that the N-terminal of most microalgae-derived antihypertensive peptides (21 out of 34 peptides) was composed of branched aliphatic hydrophobic amino acid residues, including Leu (7 peptides), Val (6 peptides), Ile (4 peptides), and Gly (3 peptides). These residues were also present at the C-terminal in 6 peptides, particularly the residues Leu, Ile, and Val. Aromatic Trp, Phe, and Tyr, and aliphatic Pro were mainly found at the C-terminal of the microalgae-derived antihypertensive peptides. Interestingly, Trp was found at the C-terminal of only the dipeptides (VW, IW, and LW) and tripeptides (TTW and VHW), and these peptides were among the most effective inhibitors against ACE, with IC_50_ ranging between 0.50 and 1.11 μM. The dipeptide VW exhibited strong ACE inhibitory activity (IC_50_ of 0.58 μM), whereas its reverse sequence WV was ~530-fold weaker as an inhibitor (IC_50_ = 307.61 μM), confirming the crucial role of Trp at the C-terminal position. Among all the ACE-inhibitory microalgae peptides, the pentapeptide LRAKA, a modified peptide from LVAKA, was the most active inhibitor, with an IC_50_ of 0.35 μM, which was ranked in the top 1% among 1246 ACE-inhibitory peptides [20]. This peptide is composed of branched aliphatic hydrophobic Leu at the N-terminal, hydrophobic Ala at the C-terminal, and positively charged Lys at the penultimate position.

At the molecular level, ACE is grouped into two distinct isoforms: somatic (sACE) and testicular (tACE), in which the former isoform is widely expressed and found on the surface of different cells, including endothelial, epithelial, neuroepithelial, and as immune cells. Therefore, sACE serves as the ACE model in most of the studies. The extracellular C-terminal of sACE comprises two homologous catalytic domains, namely the N- and C-domain, with each domain containing a zinc-binding motif-HEXXH (where X is any amino acid). Zinc ion may play a direct catalytic role either as a substrate binding site and/or catalytic site and also stabilise active site conformation. The C-domains contains two chloride ions (Cl1 and Cl2). Cl1 is located 20.7 Å from the active site zinc ion and is coordinated by Arg186, Trp485, Arg 489, and a water molecule. Cl2, which is closer to the zinc ions at 10.4 Å, is coordinated by Arg522, Tyr224, and a water molecule and plays a functional role in modulating ACE activity by stabilising the substrate within the active site [131]. Additionally, both N- and C-domains contain the following three key active pockets: (i) the S1 pocket consists of Ala354, Glu384, and Tyr523; (ii) the S2 pocket contains Gln281, His353, Lys511, His513, and Tyr520; and (iii) the S′ pocket contains only Glu162 [90]. Molecular docking studies were employed to understand the structure–activity relationships of microalgae-derived antihypertensive peptides when interacting with key enzymes/components in modulating hypertension. Microalgae-derived antihypertensive peptides interact with their targets (e.g., ACE) through various forces, including hydrogen bonds, van der Waals forces, hydrophobic interactions, salt bridges, and π-π stacking. Among them, hydrogen bonding was the most frequent and played a major role in stabilising the ligand-receptor complex. Zhang et al. [114] observed that the tripeptide VTY and tetrapeptide LGVP interacted with the active pockets of ACE mainly through multiple hydrogen bonds; VTY interacted with the active pockets S1 and S2, whereas LGVP only interacted with the S1 pocket. Also, the N-termini Val of the tripeptide and Leu of the tetrapeptide participated in coordinating Zn^2+^ ions, while the C-terminal Tyr of the tripeptide formed π-π stacking. Another study showed that the interaction of ILLYR with ACE was initiated by the amino acid residues at the tripeptide C-terminal position (Leu, Tyr, and Arg) through several hydrogen bonds, but outside the active site except for the residue Arg522 (Cl2 binding site) [124].

### 3.3. Antidiabetic Peptides

Diabetes mellitus (DM) is a global health concern, with the number of cases rising from 200 million in 1990 to 830 million in 2022. DM was reported as a direct cause of global mortality, recording 1.6 million deaths. Moreover, 530,000 kidney disease deaths and 11% of cardiovascular deaths were caused by diabetes [132]. DM is a chronic metabolic disorder characterised by high blood sugar levels (hyperglycaemia). This condition occurs when the pancreas does not produce sufficient insulin or when insulin action is impaired in the body. Insulin, a hormone produced by β-cells in the pancreas, regulates blood glucose by promoting the absorption of glucose from the bloodstream into cells for energy production. Type 1 diabetes (T1DM) and type 2 diabetes (T2DM) are the main groups of DM, with the latter accounting for 90% of all diabetes cases. T1DM (insulin-dependent) is an autoimmune condition that causes the body to attack and destroy the insulin-producing cells, causing dysfunction of the pancreas in secreting insulin. In contrast, T2DM (non-insulin dependent) is caused by the low production of insulin or the inability of cells, especially muscle and adipose tissue, to respond to the insulin produced (insulin resistance), thus lowering the glucose clearance from the blood [82,133].

**Figure 3:**
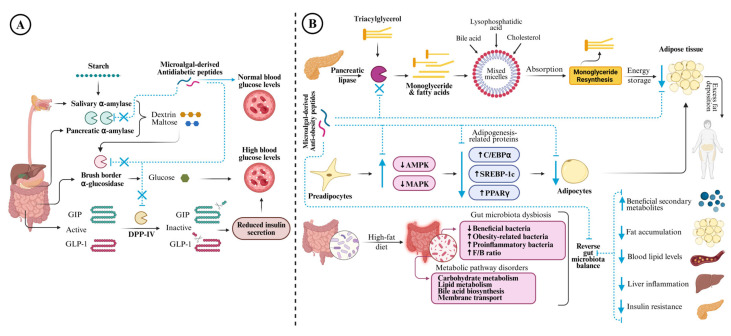
Action mechanism of microalgae-derived peptides in managing diabetes and obesity. (**A**) Microalgae-derived antidiabetic peptides inhibit key carbohydrate-degrading enzymes, including salivary and pancreatic α-amylase and brush border α-glucosidase, reducing glucose release and subsequent levels in the bloodstream. Additionally, these peptides inhibit DPP-IV, preventing the degradation of incretin hormones (GIP and GLP-1), thereby enhancing insulin secretion and contributing to the maintenance of normal blood glucose levels. (**B**) Microalgae-derived anti-obesity peptides reduce fat digestion by inhibiting pancreatic lipase, limiting the formation and absorption of monoglycerides. They also inhibit adipogenesis by activating AMPK and MAPK pathways and downregulating adipogenesis-related proteins and reducing preadipocyte differentiation. Furthermore, these peptides reverse gut microbiota dysbiosis associated with high-fat diets, restoring microbiota balance and improving obesity-related disorders (e.g., fat accumulation, dyslipidaemia, hepatic inflammation, and insulin resistance). Arrows indicate activation or direction of pathways; blue downward arrows show downregulation due to peptide action, blue upward arrows represent upregulation due to peptide action, and blue “X” indicates inhibition by peptide action. Created in BioRender. Qoms, M. (2025) https://BioRender.com/dax4wk3, accessed on 21 August 2025.

The primary source of human blood sugar originates from dietary intake, predominantly through the consumption of carbohydrates, with starch being a major component of the carbohydrate content. The carbohydrate-degrading enzyme α-amylase (salivary and pancreatic) breaks down complex starches into oligo- and disaccharides, which are further hydrolysed by α-glucosidase in the small intestine into monosaccharaides (glucose) for assimilation, where continuous glucose assimilation can increase postprandial glucose levels. Therefore, regulating the enzymatic process involving both α-amylase and α-glucosidase can reduce glucose absorption, subsequently decreasing postprandial glucose levels [82,90,134]. Additionally, a crucial enzyme known as dipeptidyl peptidase 4 (DPP-IV), which is widely distributed in the human body, degrades and inactivates incretin hormones, including glucagon-like peptide 1 (GLP-1) and glucose-independent insulinotropic polypeptide (GIP). The incretin hormones GLP-1 and GIP act by stimulating glucose-dependent insulin secretion, but they are quickly degraded by DPP-IV. Consequently, inhibiting the enzyme DPP-IV can prevent the degradation of the incretin hormones and increase their half-life, leading to increased insulin secretion and better regulation of blood glucose levels [28,84]. Inhibiting the key enzymes, α-amylase, α-glucosidase, and DPP-IV, involved in glucose metabolism is an effective approach for diabetes treatment (Figure 3A). Several enzyme inhibitors, such as acarbose, linagliptin, saxagliptin, vildagliptin, sitagliptin, voglibose, miglitol, and orlistat, are commercially available as antidiabetic drugs. Nevertheless, these synthetic drugs have been linked to several health complications such as diarrhoea, abdominal pain, flatulence, and upper respiratory tract infections [28,134,135]. Hence, natural bioactive compounds are needed as an alternative to synthetic drugs for the prevention of diabetes.

While research on antidiabetic peptides derived from microalgae is limited, these peptides have shown promising potential in modulating the activity of key enzymes linked to diabetes, including α-glucosidase, α-amylase, and DPP-IV. Villaró et al. [17] reported that *A. platensis* protein hydrolysates generated by alcalase showed higher cell-free DPP-IV inhibitory activity of 47% when tested at 1 mg/mL, compared to other proteolytic enzymes (pepsin, papain, and ficin). Another study investigated the cell-free DPP-IV inhibitory activity of *A. platensis* phycobiliprotein hydrolysates, with papain-generated hydrolysates showing the highest inhibitory activity at an IC_50_ of 3.82 mg/mL, compared to other hydrolysates produced using pepsin, trypsin, alcalase, and bromelain (IC_50_ = 4.10–5.60 mg/mL) [136]. The observed variation in both studies was due to the differences in the protein substrates used for hydrolysis, whereby the former study used the whole protein extracted from *A. platensis*, while the latter used only the phycobiliprotein (C-phycocyanin and allophycocyanin) fractions isolated from *A. platensis* proteins. Thongcumsuk et al. [28] found that *Arthrospira* sp. protein hydrolysed using trypsin exhibited moderate DPP-IV inhibitory activity at 24.46%, which was significantly increased to 74.20% (IC_50_ = 0.46 mg/mL) after peptide purification using 70% isopropanol and 0.1% trifluoroacetic acid. Additionally, peptide fractions < 3 kDa derived from a *Chlorella* mix (mixture of *Chlorella* sp. 92.50%, *Scenedesmus* sp. 5%, and *Chlorocococcus* sp. 2.50%) and *Scenedesmus* mix (mixture of *Scenedesmus* sp. 80%, *Chlorella* sp. 3%, and diatom 17%) possessed cell-free antidiabetic activity against α-amylase, with stronger inhibitory activity observed in the *Chlorella* mix (71.32%) compared to the *Scenedesmus* mix (28.78%) at 1 mg/mL [137].

A research group [52,138,139] has investigated the DPP-IV inhibitory activity of *A. platensis* and *C. pyrenoidosa* protein hydrolysates generated using pepsin or trypsin in both cell-free and Caco-2 cell models. They found that all the generated peptides displayed dose-dependent inhibitory effects with IC_50_ ranging between 0.10 and 5 mg/mL. However, the DPP-IV inhibitory activity was stronger in the cell-free assay than in the cell model when tested at the same concentrations, likely due to peptide degradation by the action of proteases produced by the Caco-2 cells. To date, only one study has investigated the in vivo antidiabetic activity of microalgae-derived peptides [84]. The generated protein hydrolysates from *C. sorokiniana* using cellulase AP3 and protease N exhibited good α-glucosidase inhibitory activity (IC_50_ = 24.50 mg/mL), while a more potent inhibitory activity against DPP-IV was indicated by a lower IC_50_ of 5.40 mg/mL. The in vivo antidiabetic effect was investigated in streptozotocin (STZ)-induced diabetic male Sprague Dawley rats fed with experimental diets containing *C. sorokiniana* protein hydrolysates only (5%) or combined with freshwater clam protein hydrolysates (2.50% + 2.50%) for a period of 4 weeks. Plasma glucose concentration in diabetic rats fed with *C. sorokiniana* protein hydrolysates was significantly reduced by 4.10%, and a more pronounced reduction of 14.6% was seen with the combined treatment. During the oral glucose tolerance test, only the combined treatment of the two different protein hydrolysates significantly reduced the plasma glucose concentration after 30 min, suggesting their synergistic effect as antidiabetic peptides. Additionally, either single or combined treatments significantly decreased concentrations of and homeostasis model assessment-estimated insulin resistance (HOMA-IR) after 4 weeks of feeding, indicating that microalgae peptides can modulate insulin resistance and improve insulin sensitivity to regulate blood glucose levels.

Several attempts have been undertaken to identify specific antidiabetic peptide sequences from microalgae protein hydrolysates and investigate their effects (Table S3). Nonetheless, the antidiabetic effects of all the reported peptides were confirmed only in cell-free assays. A total of 18 antidiabetic peptides have been derived from some species of microalgae proteins, including *Arthrospira* sp. [28], *A. platensis* [91], *C. vulgaris* [140], and *C. sorokiniana* [84]. Most of these peptides possessed strong DPP-IV inhibitory activity, with IC_50_ ranging between 102.26 and 953.00 μM, except for two peptide sequences, which showed high IC_50_ > 5000 μM, while some peptides were multifunctional antidiabetic agents, having additional inhibitory activity against α-amylase and α-glucosidase. For example, Hu et al. [91] discovered three antidiabetic peptides, RNPFVFAPTLLTVAAR, LRSELAAWSR, and GVPMPNK, from *A. platensis* exhibiting potent inhibitory activity against DPP-IV (IC_50_ = 102.26–259.50 μM), α-glucosidase (IC_50_ = 92.83–204.45 μM), and α-amylase (IC_50_ = 264.20–608.13 μM). It can be observed that the DPP-IV and α-glucosidase inhibitory activity of the three peptides was stronger than that against the α-amylase. This effect is beneficial because when the activity of α-amylase is excessively suppressed in the pancreas, can lead to abnormal bacterial fermentation of undigested polysaccharides in the colon. Therefore, moderate inhibition of α-amylase with strong α-glucosidase inhibitory activity can address issues, such as abdominal pain, diarrhoea, and flatulence associated with synthetic diabetes enzyme inhibitors.

As shown in Table S3, the structural features of microalgae-derived antidiabetic peptides reveal that they generally possess low molecular weights of <1 kDa and short chain lengths of <10 amino acid residues, except for LRSELAAWSR and RNPFVFAPTLLTVAAR derived from *A. platensis*, which exhibit relatively higher molecular weights (1187 and 1772 Da) and chain lengths (10 and 16 residues). A close inspection of the type of amino acids and their specific placement within the sequences revealed that all microalgae-derived antidiabetic peptides contain hydrophobic amino acid residues, with 94% of the peptides (17 out of 18 peptides) comprising more than 50% hydrophobic residues. The hydrophobic amino acid Pro was the most frequently occurring residue, present in 66% of the peptides (12 peptides), particularly at the second or third positions of the N-terminal. Peptides with Pro or Ala at the second position of the N-terminal are likely to function as pseudosubstrates, mimicking the substrates of DPP-IV, thus competing to bind to its active site [141]. This mechanism of competitive inhibition arises due to the structural similarity between these peptides and DPP-IV’s physiological substrates. Such an effect was found in the microalgae-derived tripeptides VPW and IPR, which inhibited DPP-IV activity through a competitive mechanism, as determined by the Lineweaver-Burk plot. Molecular docking further revealed their binding interactions via hydrogen bonds, Van der Waals forces, and hydrophobic interactions with the active pockets (S1 or S2) of DPP-IV. Additionally, these two peptides showed high gastrointestinal stability and inhibited the DPP-IV activity in mouse serum, but with higher IC_50_ (2.50–3.50 mM) compared to assays performed using purified DPP-IV [140].

In addition to the presence of Pro in the second and third positions of the N-terminal, the C-terminal also plays a significant role in the antidiabetic effect of microalgae-derived peptides. For example, the tripeptides VPW and VPA share the same sequence except for the last amino acid at the C-terminal, Trp (W) in VPW and Ala (A) in VPA. VPW exhibited stronger DPP-IV inhibitory activity, with a lower IC_50_ value (348.60 μM) compared to VPA (503.50 μM), suggesting that the presence of Trp at the C-terminal enhances DPP-IV inhibition, particularly in peptides containing an N-terminal Pro. A similar influence of terminal positioning was observed in dipeptides, although they lacked a Pro residue. For instance, the dipeptide WV and its reverse sequence VW both contain Trp, yet they exhibited different inhibitory activities. VW exhibited stronger DPP-IV inhibitory activity of 27.70% at 0.25 μg/mL (0.825 μM), whereas the reverse sequence (WV) showed nearly half the activity (12.70%). In contrast, WV was more potent against α-glucosidase, displaying almost 2-fold stronger activity (57.70%) than its reverse sequence VW (26.70%). This indicates that the presence of Trp at the C-terminal enhances DPP-IV inhibitory activity, while its presence at the N-terminal favours α-glucosidase inhibition in dipeptides.

### 3.4. Anti-Obesity Peptides

Obesity, a chronic and complex disease, is one of the most critical global health concerns. This condition is characterised by excessive accumulation of body fat and typically diagnosed using the body mass index (BMI). According to the WHO, 2.5 billion adults aged > 18 years were overweight (BMI ≥ 25 kg/m^2^) in 2022, with 890 million classified as obese (BMI ≥ 30 kg/m^2^) [142]. Obesity is associated with the progression of obesity-related disorders such as CVD, T2DM, non-alcoholic fatty liver disease (NAFLD), asthma, and others [54]. Several factors, such as genetic predisposition, unhealthy lifestyle, and high-calorie diets, are the main triggers for obesity. Another direct cause of obesity is the imbalance between energy intake and expenditure, where excessive energy is stored as triglycerides in adipose tissue. This leads to both an increase in the number of fat cells (adipogenesis) and the enlargement of existing fat cells (hypertrophy) [143]. Pancreatic lipase, a key enzyme involved in lipid metabolism, catalyses the hydrolysis of large dietary triglycerides (fats) into simpler molecules (free fatty acids and monoglycerides). Then, these small molecules form mixed micelles with bile salts, cholesterol, and lysophosphatidic acids and are further absorbed into enterocytes, where resynthesis of triglycerides takes place, and are finally stored in adipocytes [144]. Therefore, inhibiting the activity of pancreatic lipase can reduce lipid absorption, which in turn reduces obesity. Several synthetic anti-obesity drugs are commercially available, such as orlistat, phentermine/topiramate, liraglutide, and naltrexone/bupropion, which were developed based on pancreatic lipase inhibition. However, these medications are associated with multiple drawbacks, including gastrointestinal complications, psychiatric disorders, and cardiovascular events [144,145]. To overcome these limitations of synthetic drugs, natural bioactive peptides have been investigated as potential alternatives for current obesity treatments.

Peptides derived from microalgae species, including *A. platensis* and *C. pyrenoidosa*, demonstrated strong potential to be developed as natural anti-obesity treatments (Table S3). Microalgae-derived peptides alleviate obesity through multiple mechanisms, including regulation of lipid digestion (lipolysis), inhibiting differentiation of preadipocytes to adipocytes (adipogenesis), and modulating gut microbiota dysbiosis (increasing the abundance of beneficial bacteria with a concurrent decrease in obesogenic and pro-inflammatory bacteria) (Figure 3B). Otero and Verdasco-Martín [146] investigated the efficacy of peptides derived from *A. platensis* using different proteases in inhibiting fat-hydrolysing enzymes in a cell-free assay and found that all the peptide mixtures showed moderate inhibitory activities against pancreatic lipase (17.50–30.30% at 0.75 mg/mL) and cholesterol esterase (4.50–16.00% at 0.075 mg/mL). This moderate activity could be due to the use of crude peptide mixtures without any further purification and/or the low concentration of the tested samples. In another study by Fan et al. [57], protein hydrolysates derived from *A. platensis* using pepsin displayed notable inhibition of pancreatic lipase in cell-free assays, reaching 66.23%, which was further increased to 72% in the relatively low molecular weight peptide fractions of 3–5 kDa. Moreover, further purification of this fraction (3–5 kDa) using gel chromatography exhibited strong inhibitory activity towards 3T3-L1 preadipocytes (72.70–88.10%) at 1 mg/mL, surpassing that of simvastatin (52.61%). Four peptides, CANPHELPNK, NPVWKRK, NALKCCHSCPA, and LNNPSVCDCDCMMKAAR, were identified and showed anti-proliferative effects towards 3T3-L1 preadipocytes at 60.08, 46.89, 37.86, and 32.29%, respectively, at 2 mg/mL. Among these, the first 2 peptides significantly reduced the accumulation of triglycerides by 19.50, and 23.70%, respectively, at 0.60 mg/mL.

Zhang et al. [56] reported that *C. pyrenoidosa* alcalase-generated protein hydrolysate and its ultrafiltration and chromatographic fractions exhibited strong cell-free pancreatic lipase inhibitory activity ranging between 41.10 and 75.73%, demonstrating better activity than simvastatin (35.20%) but lower than orlistat (88.85%). Six peptides were identified from this hydrolysate. Among them, the best cell-free lipid-lowering effect was observed for the peptide SISISVAGGGR, which showed strong pancreatic lipase inhibitory activity (99.49%), bile salt adsorption capacity (10.49%), and cholesterol-lowering capacity (28.99%) [54]. Additionally, another peptide from the same hydrolysate (LLVVYPWTQR) inhibited the activity of pancreatic lipase by 47.95% and further demonstrated its lipid-inhibitory activity in 3T3-L1 cells, effectively reducing the accumulation of intracellular triglycerides by 27.90% at 0.60 mg/mL, comparable to simvastatin (24.10%). The peptide decreased the expression of adipogenesis-related proteins, including C/EBPα, SREBP-1c, and PPARγ, which are essential for the differentiation of preadipocytes to adipocytes. Moreover, the peptide treatment increased AMPKα signalling, and this activation is associated with energy metabolism, thus preventing fat accumulation. The altered proteins were mapped into a core network, indicating that the anti-obesity effect of the peptide LLVVYPWTQR was mediated by regulating key proteins involved in two major regulatory pathways: the AMPK signalling pathway (SREBP-1c, PPARγ, and AMPKα) and a pathway associated with NAFLD (C/EBPα, SREBP-1c, and AMPKα) [56].

In addition to the cell-free assays and cell-based models, the in vivo anti-obesity effects of peptides derived from *H. pluvialis*, *C. pyrenoidosa*, and *A. platensis* have been reported [147,148,149]. For example, Ke et al. [147] investigated the anti-obesity effect of the novel peptide KFTPAP derived from *H. pluvialis* residues using wide-type *C. elegans* strain N2 nematode model. The peptide treatment exhibited significant anti-obesity effects in high-fat-fed worms by reducing the overall fat content and triglyceride level, with the maximum effect observed at 100 µM. The peptide also reduced fat accumulation by decreasing lipid droplet size via the activation of the NHR-49/PPARα pathway, which can enhance fatty acid β-oxidation and lipolysis. Moreover, the *H. pluvialis*-derived peptide improved the fatty acid composition into a healthier lipid profile by promoting the desaturation of stearic acid to oleic acid. It also enhanced energy expenditure by increasing locomotor activity and shortening the defecation cycle, which implies that the mechanism of its anti-obesity effect is due to increased energy utilisation rather than reduced energy intake through the activation of the AAK-2/AMPK pathway. The molecular mechanism of the anti-obesity effect of the peptide KFTPAP is characterised by the activation of key fat synthesis genes fat-6 and fat-7, independent of the SBP-1/SREBP pathway. Zhao et al. [149] found that *A. platensis* peptides exerted anti-obesity effects by modulating the brain–liver axis through multiple pathways, including PPAR, adipocytokine, AMPK, NAFLD, and MAPK, resulting in a significant reduction in body weight, serum glucose, and total cholesterol in high-fat diet-fed mice, surpassing the effects of the positive drug Simvastatin. Liu et al. [54] investigated the in vivo anti-obesity effect of *C. pyrenoidosa*-derived SISISVAGGGR in high-fat diet-induced obese C57BL/6J male mice at low (100 mg/kg/d) and high (300 mg/kg/d) doses for 5 weeks and found that both concentrations exhibited anti-obesity activity, but the highest dose had more pronounced effects. The peptide treatments decreased body weight, epididymal fat weight, and heart weight index, as well as hepatic lipid accumulation, and prevented liver damage. Moreover, the peptide treatment regulated glucose metabolism, one of the obesity-related metabolic disorders, by lowering fasting glucose levels and improving insulin resistance in high-fat diet-induced obese mice. The peptide also significantly lowered serum levels of triglycerides and low-density lipoprotein (LDL), while concurrently increasing high-density lipoprotein (HDL) in high-fat diet-fed mice. In a follow-up study by the same research group [55] investigating the anti-obesity activity of the same peptide (SISISVAGGGR) at a higher concentration of 600 mg/kg/day for five weeks, it was found that the anti-obesity effect was better than at the lower doses of 100 and 300 mg/kg/day. In addition to its superior lipid-lowering effects, the peptide suppressed liver injury and hepatic inflammation triggered by the high-fat diet.

Chronic high-fat diets are associated with gut microbiota dysbiosis, which affects the host’s ability to extract energy from ingested food and store it in adipose tissue, consequently leading to metabolic dysfunction and obesity. Therefore, recent attention has shifted towards restoring the balance of gut microbiota as an effective strategy in the management of obesity and its related metabolic disorders. Beyond direct effects, bioactive peptides may also exert anti-obesity effects through the modulation of the gut microbiota and their secondary metabolites. Recent studies employed a combined approach of metagenomics and untargeted metabolomics analysis to investigate the relationship between the anti-obesity effect of microalgae-derived peptides and gut microbiota composition/functionally active metabolites. Evidence from recent studies involving anti-obesity peptide interventions derived from *C. pyrenoidosa* and *A. platensis* highlights the common alteration in gut microbiota community and associated pathophysiological consequences triggered by high-fat diets in mouse and rat models [55,150]. High-fat diet intake led to significant disruption in the gut microbiota community, characterised by a marked decrease in microbial diversity and richness, indicating a compromised and less resilient gut ecosystem. Particularly, high-fat diets increased the Firmicutes-to-Bacteroidetes ratio, which is a common indicator for obese individuals. Additionally, the gut microbiota displayed a reduction in beneficial bacteria phyla such as Bacteroides, Verrucomicrobia, and Proteobacteria, with a concurrent increase in obesity-associated genera such as *Ruminococcus*, *Dorea*, and *Evtepia gabavorous*. Moreover, gut microbiota dysbiosis was accompanied by functional impairments in key metabolic pathways, including those linked to carbohydrate and lipid metabolism, bile acid biosynthesis, and membrane transport. Interestingly, the peptide SISISVAGGGR derived from *C. pyrenoidosa* and phycobiliprotein-derived peptides from *A. platensis* have shown notable potential in mitigating obesity by restoring the gut microbiota abundance and diversity in high-fat diet animal models. The microalgae-derived anti-obesity peptides reversed gut microbiota dysbiosis by reducing the Firmicutes-to-Bacteroidetes ratio and increasing beneficial bacteria, such as *Bacteroides*, *Parabacteroides*, *Akkermansia muciniphila*, *Faecalibacterium prausnitzii*, *Lactobacillus johnsonii*, and *Romboutsia*, while suppressing obesogenic and pro-inflammatory bacteria such as *Dorea*, *Evtepia gabavorous*, and *Ruminococcus gnavus*. Moreover, functional and metabolomics analysis revealed that this shift in the gut microbiota was linked to improved bile acid metabolism, increased production of beneficial secondary metabolites, such as SCFAs, DL-arginine, and N-stearoyl GABA, and enhanced regulation of pathways involved in carbohydrate metabolism, amino acid metabolism, lipid metabolism, glucose homeostasis, and insulin signalling. Taken together, microalgae-derived peptides exert anti-obesity effects not only through direct metabolic regulation but also via gut microbiota-mediated mechanisms, highlighting their potential as functional dietary agents in obesity management.

The structure–activity relationship of microalgae-derived anti-obesity peptides has not been widely studied, with a limited number of peptides having been chemically synthesised and tested in either cell-free or in vivo models. Table S3 shows that only 11 anti-obesity peptide sequences have been identified in microalgae protein hydrolysates, and their activities were validated in wet experiments. Their chain length and molecular weight differ from those of other microalgae-derived bioactive peptides. Unlike antioxidant, antihypertensive, and antidiabetic peptides derived from microalgae, the anti-obesity peptides feature a relatively longer peptide chain length and higher molecular weight, ranging between 6 and 17 amino acid residues and 659–1871 Da, with most of them (8 sequences) having ≥10 residues and 1000 Da. Additionally, all microalgae anti-obesity peptides contain at least one hydrophobic amino acid residue (Pro, Ala, Leu, Gly, Val, Ile, Met, Phe, and Trp), with hydrophobic residues accounting for about 33–66% of their total amino acid composition. Particularly, hydrophobic residues including Pro, Ala, Leu, Gly, and Val were the most frequently occurring in the peptide sequences. The presence of hydrophobic amino acid residues in the peptide sequences facilitates their interaction with hydrophobic targets and enhances their permeation through the cell membrane, thereby contributing to their anti-obesity effects in living cells. Another structural feature of the microalgae-derived anti-obesity peptides is the presence of basic amino acid residues, including Lys, Arg, and His, which were found in all the peptides, with a frequency ranging from 9 to 42% of the total amino acids in their sequences. The presence of Lys and Arg was mainly located at the C-terminal of the peptides, which could influence their interaction with target molecules and tissues due to their positive charge at physiological pH. Moreover, molecular docking studies revealed that microalgae-derived anti-obesity peptides can interact with catalytic sites (Ser153, Asp177, and His 264) of pancreatic lipase through hydrogen bonding [56,57]. Nonetheless, more comprehensive investigations are needed to elucidate the molecular interactions of microalgae-derived peptides to gain deeper insights into their structure–activity relationships.

### 3.5. Anti-Ageing Peptides

Ageing is a complex and multifactorial biological process characterised by the progressive decline in physiological integrity leading to impaired function and increased vulnerability to diseases. This condition is caused by intrinsic factors such as telomere shortening and genomic instability and extrinsic factors including ultraviolet (UV) radiation, environmental toxins, and poor lifestyle habits. These factors contribute to the deterioration of tissue structure and function across various organs, with visible signs most apparent in the skin. In this context, both intrinsic and extrinsic ageing play a key role in skin ageing, with a major contribution caused by prolonged exposure to UVB radiation (photoageing), leading to increased accumulation of ROS that triggers oxidative stress, inflammation, DNA damage, and degradation of extracellular matrix (ECM) proteins such as collagen and elastin that are crucial for maintaining skin texture and elasticity [30,151,152].

Recent studies have identified several bioactive peptides derived from the microalgae *I*. *zhanjiangensis* [151,152,153], *A*. *platensis* [92,102,154,155], *H*. *pluvialis* [156], and *Synechococcus* sp. VDW [157] exhibiting promising anti-ageing effects through cell-free, cellular, and in vivo models (Table S4). The microalgae-derived peptides exerted their anti-ageing effects through multiple mechanisms, including antioxidant, anti-inflammatory, anti-apoptosis, inhibition of key enzymes involved in ECM degradation (collagenase and elastase) and pigmentation (tyrosinase), promotion of collagen synthase, and regulation of signalling pathways involved in ageing. Overexposure to UV radiation, especially UVB, increases the expression of ageing-related enzymes, thereby disrupting tissue remodelling [158]. The increased activity of collagenase and elastase can degrade dermal ECM collagen (particularly type I and type III procollagen) and elastin, respectively, consequently compromising the elasticity of the tissue. Also, hyaluronidase is another crucial enzyme that breaks down hyaluronic acid, a compound crucial for maintaining skin moisture; when overexpressed, it leads to the formation of wrinkles. Additionally, tyrosinase, a polyphenol oxidase family, catalyses melanin synthesis in melanocytes through the hydroxylation of monophenols (monophenolase activity) or the conversion of diphenols into quinone derivatives (diphenolase activity) [159,160]. Cell-free investigations showed that *A. platensis*- and *C. vulgaris*-derived protein hydrolysates containing low-molecular-weight peptides demonstrated strong anti-ageing effects by inhibiting the activity of ageing-related enzymes, including elastase (79.25 and 84.43%), collagenase (86.21 and 90.52%), and tyrosinase (58.22 and 66.12%) [30]. Additionally, *Nannochloropsis* sp. (strain NNX1) protein hydrolysates exhibited potent anti-elastase (IC_50_ = 0.026 µg/mL) and anti-hyaluronidase (IC_50_ = 0.260 µg/mL) activity [158]. In another study, Kose and Oncel [155] identified seven bioactive peptides from *A. platensis* protein hydrolysates and investigated their cell-free tyrosinase inhibitory activity (monophenolase and diphenolase) and cellular diphenolase inhibitory activity in melanoma cells. Among the tested peptides, both SPSWY and AADQRGKDKCARDIGY demonstrated strong cell-free monophenolase inhibitory activity (IC_50_ = 12.10 and 191.40 µM), cellular tyrosinase inhibitory activity (IC_50_ = 48.90 and 34.20 µM), and melanin synthesis inhibition by 26.00% and 54.80% at 200 µg/mL. However, the peptide ryvtyavf exhibited the opposite effect, increasing melanin synthesis up to 3-fold, making it a potential pigmentation agent.

In addition to the cell-free anti-ageing effects, microalgae-derived peptides also showed strong protective effects against UVB-induced damage in human keratinocyte (HaCaT) cells (epidermal skin cells) and H_2_O_2_-induced senescence in human dermal fibroblasts (BJ cells) (dermal skin cells). For instance, *I. zhanjiangensis*-derived peptides, namely DAPTMGY [152] and AYAPE [153], demonstrated potent anti-ageing effects in UVB-induced human keratinocytes via multiple mechanisms, including antioxidant action by scavenging intracellular ROS and upregulating the expression of antioxidant enzymes such as SOD, CAT, and GPx. These peptides also inhibited UVB-induced apoptosis by downregulating pro-apoptotic proteins Bax caspases (−3/−8/−9), while concurrently upregulating the anti-apoptotic protein Bcl-2 and preventing nuclear translocation of p53 and DNA damage. Moreover, the peptides regulated matrix remodelling by decreasing the secretion of collagen-degrading enzymes (metalloproteinases; MMP-1/MMP-3) while increasing procollagen I, which supports collagen synthesis and skin integrity. The peptides were found to suppress photoageing by inhibiting inflammatory pathways, including MAPK and NF-κB signalling. Another peptide, EMFGTSSET, derived from *I. zhanjiangensis*, demonstrated broad efficacy by restoring oxidative balance (ROS level) via the Nrf2/HO-1 signalling pathway, promoting autophagy via the AMPK/mTOR signalling pathway, and enhancing collagen synthesis in H_2_O_2_-induced senescence in human dermal fibroblasts through the TGF-β1/Smad axis, with comparable anti-ageing effects to vitamin C [151].

The anti-ageing effects of peptides derived from *H. pluvialis* (KFTPAP) and *A. platensis* (Ac-GMCCSR-NH_2_) have been investigated in different in vivo models. He et al. [156] found that the peptide Ac-GMCCSR-NH_2_ exhibited strong anti-ageing effects in *C. elegans* nematodes (normal and stress-induced models). This peptide extended the lifespan of the nematodes under both oxidative and thermal stress conditions and improved their physiological functions by reducing ageing pigments (lipofuscin) and enhancing locomotion and pharyngeal pumping activity without impairing reproduction. This effect was related to the antioxidant mechanism of the peptide by enhancing the activity of antioxidant enzymes (SOD and CAT) and upregulating gene expressions (sod-3 and ctl-2). Interestingly, the insulin/insulin-like growth factor signalling (IIS) pathway was critical for the peptide’s anti-ageing effect rather than the MAPK pathway, particularly through the regulation of the subcellular localisation of DAF-16. In another study, the peptide Ac-GMCCSR-NH_2_ possessed potent anti-photoageing effects in UVB-irradiated mice by normalising epidermal thickness, restoring skin moisture, reducing inflammation, and vacuolar degeneration (morphological observation). Additionally, the peptide increased antioxidant enzyme activity while reducing the levels of collagen-degrading enzymes (MMP-1/MMP-3) and MDA in skin tissue of irradiated mice. Proteomic analysis further revealed the impact of the peptide on multiple cellular processes, including keratinisation and glycolysis pathways. Interestingly, this peptide (Ac-GMCCSR-NH_2_) possessed a safer profile in HaCaT cells and better anti-photoageing activity compared to the commercial drug Matrixyl [154].

Overall, microalgae-derived peptides demonstrated potent anti-ageing effects in different models, with some peptides being comparable or even superior to some commercially available anti-ageing compounds such as vitamin C and Matrixyl, making them a promising candidate to be developed as anti-ageing treatments, particularly for anti-photoaging applications.

As shown in Table S4, structural features of microalgae-derived anti-ageing peptides (14 peptides) revealed that they are low molecular weight (<2 kDa) with a short chain length of 3–16 residues. Masoumifeshani et al. [30] found that lower molecular weight peptide fractions (<3 kDa) obtained from both *A. platensis* and *C. vulgaris* protein hydrolysates were more effective inhibitors against ageing-related enzymes (elastase, collagenase, and tyrosinase) compared to higher molecular weights (3–10 and >10 kDa). Additionally, all the peptide sequences, except DER, contained hydrophobic amino acid residues, particularly Gly, Ala, Phe, Pro, Met, and Leu, with 50% of the peptides (7 out of 14 sequences) having more than 50% hydrophobic residues. Additionally, aromatic amino acids, particularly Phe and Tyr, were found in 71% (10 out of 14) of the peptide sequences. The role of some of these residues in the anti-ageing effect was confirmed using a molecular docking study of the peptide AILESYSAGKTK derived from *Synechococcus* sp., which competitively interacted with tyrosinase, with the residues Tyr, Ala, Leu, and Ile contributing to the hydrophobic interactions that stabilised the peptide within the tyrosinase active site [157]. We also found that about 71% (10 peptides) of the microalgae-derived peptides contained basic amino acids, including Lys and Arg, while acidic amino acids (Asp and Glu) were present in only 43% of all the peptides (6 sequences). The sulphur-containing amino acids Cys and Met were found in 43% of all the peptides (6 sequences). On the other hand, hydrophobic amino acids were the most frequently found residues at both ends (N-/C-terminal) of the microalgae anti-ageing peptides. In addition, the aromatic residues Phe and Tyr were found at the C-terminal of most sequences (5 peptides). The presence of Tyr at these positions within the peptide sequence may enhance their anti-ageing effects by enabling specific interactions with tyrosinase. This is because the phenolic hydroxyl group of Tyr can mimic the natural substrates of tyrosinase, allowing the peptides to act as a competitive inhibitor and reduce melanin production [155]. Moreover, Tyr contributes to the peptide’s antioxidant activity by donating hydrogen atoms to neutralise free radicals [152]. Among the seven anti-ageing peptides derived from *A. platensis*, C-terminal Tyr-containing peptides (SPSWY and AADQRGKDKCARDIGY) were among the most active anti-ageing peptides with anti-tyrosinase inhibitory activity [155]. Also, *I. zhanjiangensis*-derived DAPTMGY with C-terminal Tyr showed a potent anti-ageing effect against UVB-induced human keratinocyte cells [152]. This observation is supported by the commercially available anti-ageing peptide YRSRKYSSWY, which contains Tyr at both ends, exhibiting strong and competitive anti-tyrosinase inhibitory activity (IC_50_ = 40 µM) [161]. The observed structural features of the microalgae-derived anti-ageing peptides were closely correlated with their antioxidant activity (as discussed in Section 3.1). This alignment is anticipated, as the antioxidant mechanism is a central pathway underlying the anti-ageing effects of the microalgae-derived peptides.

### 3.6. Other Bioactive Peptides Derived from Microalgae

While most research on microalgae peptides has centred around antioxidant, antihypertensive, antidiabetic, anti-obesity, and anti-ageing activities, recent studies have uncovered additional, yet less extensively explored, bioactivities. These bioactive peptides include anticancer [53,101], anti-inflammatory [31,97,98,162], anti-osteopenic [163], calcium-binding [164,165], wound-healing [99,166], and antimicrobial [32,167,168,169,170,171] properties.

For example, the *A. platensis*-derived peptide (YGFVMPRSGLWFR) demonstrated potent and dose-dependent anticancer activity against different cell lines, including colon cancer cells (HT-29), lung cancer cells (A549), hepatoblastoma cells (HepG-2), and gastric cancer cells (SGC-7901), with an IC_50_ ranging between 104.05 and 446.72 µg/mL, while the peptide showed minimal cytotoxicity against immortalised liver cells (L-o2) [53]. Another peptide (GGTCVIRGCVPKKLM) derived from the same microalga possessed strong anticancer activity when investigated in oral carcinoma cells (KB) at 25 μM by inducing caspase-9-mediated apoptosis, membrane disruption, and DNA degradation, and showed a comparable effect to the positive drug doxorubicin (2 μM) [101]. Additionally, *I. zhanjiangensis*-derived peptides demonstrated both anti-angiogenic and anti-inflammatory activities, such as the peptide IIAVEAGC [97] exerting its effects by inhibiting the production of ROS and pro-inflammatory factors through the Nrf2/SOD/HO-1 and NF-κB signalling pathways and suppressing angiogenesis by reducing the expression of matrix metalloproteinases (MMP-2 and MMP-9) via the PI3K/AKT, NF-κB, and MAPK pathways. Another peptide, IIAVE, truncated from IIAVEAGC, exhibited neuroprotective effects in a 6-OHDA-induced Parkinson’s model by reducing oxidative stress and restoring mitochondrial potential [172].

Calcium-binding peptides derived from *Schizochytrium* sp. have been recently reported by Cai et al. [164,165], with two dipeptides, FY and YL, significantly increasing the capacity of calcium binding by 128.77 and 126.34 μg/mg, forming stable chelates with calcium (FY-CA and YL-CA) via carboxyl and amino groups. Both FY-CA and YL-CA enhanced calcium uptake efficiency by >3-fold compared to CaCl_2_ in Caco-2 cells and protected calcium ions against dietary inhibitors (oxalate, tannic acid, phytate, and zinc).

Antimicrobial peptides have also emerged from diverse microalgae species, including *A. platensis* [168], *Aureococcus anophagefferens* [32], *Thalassiosira oceanica* [169], *Limnospira maxima* [170], *Tetraspora* sp. [167], and *Tetraselmis suecica* [171]. These peptides exhibited antibacterial activity against Gram-positive bacteria, Gram-negative bacteria, and fungi, with minimal haemolytic and cytotoxicity effects towards red blood cells and normal cells. Research on the mechanism and structure–activity relationship of microalgae-derived antimicrobial peptides is still limited. However, Zhang et al. [32] reported a 12-amino acid peptide (RKLLRVIKDLIK) derived from *A. anophagefferens*, carrying a cationic charge (+4) and adopting an α-helical conformation. This peptide possessed potent broad-spectrum activity with minimum inhibitory concentration (MIC) values of 31.25 µg/mL against *E. coli*, 125 µg/mL against *S. aureus* and *M. luteus*, and 62.5 µg/mL against *Pichia pastoris*. The amphipathic helical structure, with hydrophobic amino acid residues (a common feature of antimicrobial peptides) aligned on one face of the helix, can facilitate pore formation in the cell membrane through the barrel-stave model or toroidal pore model. Scanning electron microscopy confirmed that this helical peptide disrupted the bacterial membrane, producing holes that led to intracellular leakage. Similarly, Guzmán et al. [171] identified several antimicrobial peptides from *T. suecica*, including AQ-1756 (random coil), AQ-1757 (β-turn), and AQ-1766 (helical tendency). Among these peptides, AQ-1766 exhibited the strongest antimicrobial activity against both Gram-positive and Gram-negative bacteria, supporting the notion that an α-helical structure is favourable for the activity of microalgae-derived antimicrobial peptides. Alanine scanning further demonstrated the importance of aromatic residues, as their substitution disrupted helicity, which markedly reduced the antimicrobial effect, while lysine substitutions increased the cationic charge, stabilised the helical structure, and enhanced the antimicrobial activity. These findings highlight that microalgae-derived antimicrobial peptides often possess an amphipathic helical structure enriched in cationic and aromatic residues, exerting their antimicrobial effect through a membrane disruption mechanism.

These functions, though less reported, suggest broader physiological roles of microalgae-derived peptides, which broaden their application in functional foods, nutraceuticals, and pharmaceuticals. Nevertheless, more comprehensive investigations are essential, particularly in vivo studies and detailed analysis of their action mechanisms and structure–activity relationships, to validate their efficacy and support their practical development in different sectors.

## 4. Structural Modification of Microalgae-Derived Peptides for Enhanced Bioactivities

Modifications of bioactive peptides can enhance their bioactivities and gastrointestinal stability, thereby increasing their potential use as therapeutic agents. Various modification approaches, such as N/C-terminal substitution/modification, D-amino acid substitution, cyclisation, amino acid scanning (such as Ala, Pro, Gly, Leu, and Val), and truncation, have been extensively explored in peptide research [173,174].

Peptide modifications using N-terminal acetylation and C-terminal amidation are commonly used to neutralise the charges at both ends of peptides, thereby mimicking natural peptides. Such modifications can improve peptide stability by decreasing their susceptibility to enzymatic degradation by aminopeptidases and carboxypeptidases that cleave at the N- or C-terminal of peptides. Additionally, this approach can enhance the binding affinity of bioactive peptides to target receptors and enzymes in biological systems. Moreover, acetylation and amidation of peptide termini can improve their binding to the cell membrane, thus enhancing their permeability [175,176]. This approach was successfully employed to modify the *A. platensis*-derived peptide GMCCSR, whereby the acetylated and amidated peptide (Ac-GMCCSR-NH_2_) exhibited potent anti-ageing effects in ultraviolet B-irradiated keratinocytes and mice and was superior to the positive control Matrixyl [154]. Min et al. [169] also investigated the effect of N-terminal acetylation and C-terminal amidation of the antimicrobial peptide RRRRGGRGRGRRRGRG derived from *Thalassiosira oceanic*. The modified peptide (Ac-RRRRGGRGRGRRRGRG-NH_2_) possessed a 4-fold stronger antibacterial effect against Gram-positive bacteria (*S. aureus* and *B. Subtilis*) and 2- to 4-fold stronger antibacterial effect against Gram-negative bacteria (*E. coli* and *P. aeruginosa*), compared to the original peptide sequence.

Amino acid scanning is a sequential substitution of individual amino acid residues in a peptide sequence, which enables the determination of the contribution of a specific residue to the bioactivity. Amino acid residues such as alanine, leucine, proline, glycine, and valine are used in this technique for peptide modification, in which alanine scanning is the most common approach. This is due to the presence of the chemically inert and non-bulky methyl side chain in the alanine structure that does not change the secondary structure of a peptide. This approach is often coupled with saturation mutagenesis by replacing the least crucial amino acid residues that are not critical to the peptide’s biological activity [173]. For instance, Suo et al. [20] used alanine scanning and saturation mutagenesis (combined with in silico and cell-free analyses) using the residue scanning module in Schrödinger for modifying and optimising the ACE-inhibitory activity of LVAKA discovered from *C. pyrenoidosa*. The amino acid residues, including Leu1 and Lys4, were critical residues for maintaining the ACE inhibitory activity of the peptide; therefore, the residues Val2, Ala3, and Ala5 were mutated based on empirical principle. A total of 14 modified peptides with mutated amino acid residues were selected based on binding energy with ACE and synthesised to further investigate their effect on the ACE activity. Interestingly, among all the modified peptides, LRAKA (Val2 substituted with Arg) and LRAKP (Val2 substituted with Arg and Ala5 substituted with Pro) exhibited the most potent cell-free ACE inhibitory activity with IC_50_ at nanomolar levels of 350 nM and 930 nM, respectively, representing a 28- and 76-fold reduction in the IC_50_ of the original peptide (LVAKA; IC_50_ = 26.66 μM). Additionally, the antihypertensive effect of LRAKA was investigated in vivo using the SHR model and successfully lowered systolic and diastolic blood pressure when administrated intraperitoneally at 20 mg/kg body weight, comparable to the positive drug Captopril. Guzmán et al. [171] also used an alanine scanning approach followed by lysine substitution to modify the antibacterial peptide LWFYTMWH obtained from *Tetraselmis suecica*. Alanine scanning was used to substitute each residue at a time to identify the most critical residues within the peptide sequence, demonstrating that Ala substitution at positions 1, 4, 5, and 6 improved the antibacterial effect of the original peptide. Subsequently, individual substitution of Ala residues in the improved peptide analogues (obtained from the alanine scanning) with Lys residues further enhanced the antibacterial effect towards both Gram-positive and Gram-negative bacteria, particularly *S. typhimurium*, *B. cereus*, and *S. aureus*, with a 2- to 5-fold reduction in their minimum bactericidal concentrations.

Peptide truncation is another modification approach that has been investigated to enhance the bioactivity of microalgae-derived peptides. Peptide truncation refers to the process of shortening a peptide length by removing one or more amino acid residues from the N- or C-terminal regions of the original peptides [177]. This modification approach aims to enhance the desired bioactivity and bioavailability of a peptide, as shorter peptides are often less susceptible to proteolytic degradation and more easily absorbed by the body. A recent study by Lin et al. [94] employed truncation of the peptide IIAVEAGC by performing the following: (i) removing the last three residues at the C-terminus, resulting in a pentapeptide with the sequence IIAVE and (ii) removing the first five residues at the N-terminus, resulting in a tripeptide with the sequence AGC. The original and truncated peptides were tested for their cellular antioxidant activity in an H_2_O_2_-induced oxidative stress model, in which the truncated peptide IIAVE exhibited stronger cytoprotective effects, although the other two peptides remained active.

It is evident that modification and optimisation of microalgae-derived peptides have led to the discovery of novel and potent biologically active sequences, particularly when modification approaches were assisted by in silico analysis. As discussed earlier, the modified nanomolar-level ACE-inhibitory peptide LRAKA was discovered from the original sequence LVAKA, with a 76-fold stronger inhibitory activity [20]. This novel peptide is ranked among the top 1% of ACE-inhibitory peptides and can substitute commercial drugs such as Captopril. These findings serve as a benchmark for more comprehensive studies exploring other modification techniques such as cyclisation, D-amino acid substitution, and conjugation with other molecules like PEG, which have not been investigated for microalgae peptides, to discover more powerful bioactive peptides.

## 5. Conclusions and Future Perspective

Microalgae are a promising protein-rich substrate, with numerous species having GRAS status, making them a good source of natural bioactive peptides. Microalgae-derived bioactive peptides demonstrate low molecular weight with a wide range of biological activities that enhance their bioavailability in the human body, thereby enabling their application in the field of food and medicine. This paper provides a comprehensive review of the current research on the production and discovery of microalgae-derived peptides, their roles and molecular mechanisms in various biological activities, their structure–activity relationships, as well as their structural modifications for enhanced biological efficacy. While enzymatic hydrolysis is one of the most widely applied techniques for the generation of bioactive peptides from microalgae, the hydrolysis process must be standardised and carefully monitored in order to obtain the desired activity with consistent outcomes. In addition, the proteases used in the cleavage of microalgae proteins are expensive; therefore, more research on the use of immobilised enzyme techniques for the production of bioactive peptides is essential to enable their large-scale production at a lower cost. Alternatively, bioinformatics and computational tools, including in silico enzymatic hydrolysis, QSAR, and molecular docking, have emerged as powerful approaches in the preparation and discovery of microalgae peptides with potent bioactivity, which is expected to be a future trend in peptide research as it is rapid and more cost-effective compared to conventional techniques. Furthermore, microalgae-derived peptides exhibit various biological activities, including antioxidant, antihypertensive, antidiabetic, anti-obesity, and anti-ageing effects, mediated through diverse mechanisms such as intracellular signalling modulation and enzyme/protein inhibition. Also, analysis of the structural features, such as molecular weight, chain length, type of amino acids, and their specific placement within sequences, provides insights into their structure–activity relationships. However, investigations into the relationship between the secondary structure of microalgae-derived peptides and their activity remain limited to a few antioxidant and antimicrobial studies. Future research employing techniques such as circular dichroism and in silico tools across a wider range of bioactivities is needed to establish a more comprehensive framework linking peptide conformation to biological performance. By synthesising structure–activity relationship insights from microalgae-derived peptides, this review underscores how rational peptide design can be guided by a combination of empirical evidence and computational modelling, thus enabling large-scale production of bioactive peptides with potent activities. Nevertheless, other microalgae-derived bioactive peptides, such as those with anticancer, anti-inflammatory, anti-osteopenic, calcium-binding, wound-healing, and antimicrobial properties, have received little attention in the literature, therefore further studies are warranted to fully elucidate their biological activities and mechanisms of action. Moreover, it is important to note that current studies on the biological activities of microalgae-derived peptides have primarily relied on chemical-based assays as well as cell and/or animal models. Therefore, future efforts should aim to advance these findings toward clinical application by considering crucial factors such as stability, delivery, and immunogenicity, which are essential for their clinical and industrial translation. Peptides are prone to enzymatic degradation in the gastrointestinal tract, leading to a short half-life and poor bioavailability. To address this challenge, novel delivery systems such as nanoparticles or nanoconjugates can be developed to encapsulate and stabilise microalgae-derived peptides. Although these peptides exhibit promising bioactivity in vitro and in animal models, well-designed clinical trials are still lacking to establish their long-term safety, efficacy, and pharmacokinetic profiles in humans. Addressing these issues will be essential to translate microalgae-derived peptides from laboratory findings into clinically viable peptide-based therapeutics.

## Supplementary Materials

The following supporting information can be downloaded at: https://www.mdpi.com/article/10.3390/antiox14101170/s1, Table S1: Antioxidant peptides derived from different microalgae species discovered in the past ten years; Table S2: Antihypertensive peptides derived from different microalgae species discovered in the past ten years; Table S3: Antidiabetic and anti-obesity peptides derived from microalgae discovered in the past ten years; Table S4: Anti-ageing peptides derived from different microalgae species discovered in the past ten years.
