# Supplementary material for: Microalgae-Derived Peptides Targeting Lifestyle-Related Diseases: Discovery, Mechanisms, Structure–Activity Relationships, and Structural Modifications
Source: Antioxidants (Basel). 2025 Sep 25;14(10):1170. doi: 10.3390/antiox14101170 (PMC12561970; doi:10.3390/antiox14101170)
Supplement: Supplementary file 1 [file antioxidants-14-01170-s001.zip › antioxidants-3854688-supplementary.pdf]

Microalgae-Derived Peptides Targeting Lifestyle-Related Diseases: Discovery, Mechanisms, Structure-Activity Relationships and Structural Modifications

Mohammed S. Qoms[1], Sok Kuan Wong[2], Norsyahida Mohd Fauzi[3], Khairana Husain[3], Suzana Makpol[1], Jen Kit Tan[1,*]

[1]Department of Biochemistry, Faculty of Medicine, Universiti Kebangsaan Malaysia, Jalan Yaacob Latif, Bandar Tun Razak, 56000 Cheras, Kuala Lumpur, Malaysia.

[2]Department of Pharmacology, Faculty of Medicine, Universiti Kebangsaan Malaysia, Jalan Yaacob Latif, Bandar Tun Razak, 56000 Cheras, Kuala Lumpur, Malaysia.

[3]Centre for Drug and Herbal Development, Faculty of Pharmacy, Universiti Kebangsaan Malaysia, Jalan Raja Muda Abdul Aziz, 50300 Kuala Lumpur, Malaysia.

***Corresponding author**

Jen Kit Tan

E-mail: jenkittan@ukm.edu.my

Table S1: Antioxidant peptides derived from different microalgae species discovered in the past ten years

Peptide source/sequence	MW (Da)	PCL	Assay model/ Effective concentration	Key findings/mode of action	Ref.
			**Cell-free & molecular docking**		
Haematococcus pluvialis					
GVFHPGPF<br>IDPGTWRPL<br>APIIGKPAPGFK	856<br>1053<br>1194	8<br>9<br>12	DPPH, ABTS, HRS, FRAP<br>0.33- 1.10 mg/mL<br>0.14- 0.89 mg/mL<br>0.25- 0.78 mg/mL	- Strong radical scavenging and reducing power activity<br>- Interacted with Keap1 through hydrogen bonds and hydrophobic interactions<br>- interacted with DPPH and ABTS via hydrogen bonds	[105]
Auxenochlorella pyrenoidosa					
AGWACLVG<br>YPLDL<br>IDLAY	776<br>620<br>594	8<br>5<br>5	DPPH<br>200 µg/mL	- Strong DPPH radical scavenging activity ranging between 81.11-89.31%<br>- IC_{50} = 53.45 µg/mL (68.88 µM) only reported for AGWACLVG	[89]
Schizochytrium limacinum					
PYK	406	3	DPPH, HRS<br>0.10-1.00 mg/mL	- Comparable radical scavenging to the positive control L-glutathione (GSH)	[110]
Arthrospira platensis					
VTAGLVGGGAGK	986	12	ABTS, HRS, FIC<br>1.08-1.35 mg/mL	- Highly stable radical scavenging under thermal processing (25-100°C) and pH conditions (pH 3-11) as well as during *in-vitro* gastrointestinal digestion	[48]
***Synechococcus* sp. VDW**					
AILQSYSAGKTK<br>ALNKTHLIQTK<br>IPDAHPVK<br>LLVHAPVK<br>VVVLRDGAVQQLGTPR	1266<br>1266<br>876<br>876<br>1708	12<br>11<br>8<br>8<br>16	NO<br>200 µg/mL	- Moderate NO scavenging activity (10.38-20.89%).	[111]
Tetradesmus obliquus					
WPRGYFL<br>SDWDRF	937<br>824	7<br>6	DPPH, ABTS<br>4.70-13.97 µM	- Dose-dependent radicals scavenging activity with relatively low IC_{50}.	[109]
			**Cell model**		
Arthrospira platensis					
INSSDVQGKY	1109	10	H_2O_2-stressed HaCaT cells<br>100-400 µg/mL	- Good *in-vitro* ORAC (0.32 mmol Trolox equivalent/g)<br>↓ ROS levels in HaCaT cells<br>Non-toxic to HaCaT cells	[49]
NPLSTQDDVAASL<br>LGLDVWEHAYYL<br>GGTCVIRGCVPKKLM	1330<br>1478<br>1561	13<br>12<br>15	H_2O_2-stressed leucocytes<br>7.8-250 µM<br>6.25-200 µM<br>6.25-200 µM	- Dose-dependent radical scavenging activity (DPPH, ABTS, HRS, SARS and NO)<br>↓ ROS production in the leucocytes<br>- Non-toxic to human blood and Vero cells	[99–101]
EYFDALA<br>GMCCSR	828<br>655	7<br>6	AAPH-stressed erythrocytes<br>1.2-120 µM<br>1.5-150 µM	- Dose-dependent DPPH (IC_{50}=5.80 & 16.94 µM) and ABTS (IC_{50} 13.40 & 36.93 µM) radical scavenging activity, comparable to the positive control vitamin C<br>↓ Oxidative haemolysis of erythrocytes<br>↓ Lipid peroxidation (MDA)<br>↑ Antioxidative enzymes (SOD, CAT, GPx)<br>- Non-toxic to human erythrocytes and L929 cells	[96,102]
Isochrysis zhanjiangensis					
IIAVEAGC<br>IIAVE<br>AGC	774<br>543<br>249	8<br>5<br>3	H_2O_2-stressed SH-SY5Y cells<br>20-100 µM	↓ ROS and mitochondrial damage<br>↑ Antioxidant enzymes (HO-1 and SOD)<br>↑ Nrf2 expression pathway<br>↓ PI3K, MAPK and NF-κB signalling pathways<br>- Non-toxic to SH-SY5Y cells	[94]
AYAPE	549	5	H_2O_2-stressed HUVEC and SH-SY5Y cells<br>20-100 µM	↑ Antioxidant enzymes (HO-1 and GPx1)<br>↑ Anti-apoptotic (↓ expression of P53 and cytochrome c proteins)<br>↑ Anti-inflammatory (↓ pro-inflammatory factors IL-6 and IL-1β)	[107]

				↓ NF-κB and MAPK signalling pathways (↓ phosphorylation of JNK and P38 proteins, and ↓ P65, P50 and Iκβ-α)<br>↑ Nrf2/Keap1 pathway<br>- Non-toxic to HUVEC and SH-SY5Y cells	
DAPTMGY	753	7	LPS-stressed HUVEC<br>1-50 μM	↓ Mitochondrial oxidative stress<br>↓ ROS production<br>↑ Antioxidative enzymes (SOD, GPx, HO-1)<br>↓ Inflammatory proteins (COX-2/ iNOS)<br>↓ NF-κB and MAPK signalling pathways<br>- Non-toxic to HUVEC cells	[108]
NDAEYGICGF	1088	10	Ethanol-stressed HepG2 cells<br>10-100 μM	↓ ROS production<br>↑ Antioxidant enzyme SOD<br>↓ GGT<br>- Non-toxic to HepG2 cells	[59]
Chlorella pyrenoidesa					
AGYSPIGFVR<br>VLDELTLAR<br>LFDPVYLFDQG	1066<br>1029<br>1313	10<br>9<br>11	AAPH-stressed HepG2 cells<br>0.04 mg/mL<br>0.09 mg/mL<br>0.41 mg/mL	↓ ROS production<br>Non-toxic to HepG2 cells	[87]
			In-vivo		
Haematococcus pluvialis					
KFTPAP	659	6	*Caenorhabditis elegans* (Nematode)<br>100 μM	↑ Antioxidant enzymes SOD, CAT<br>↓ Lipid peroxidation (MDA)<br>- Non-toxic to nematodes	[106]
Arthrospira platensis					
VKYVSPTCGPCH	1290	12	H_2O_2-stressed zebrafish and cardiomyocytes cells<br>6.25-200 μM	- Dose-dependent *in-vitro* radical scavenging activity (DPPH, ABTS, HRS and SARS)<br>↓ ROS production in zebrafish and cardiomyocytes cells<br>- Non-toxic to human blood and H9c2 cells	[103]
Arthrospira platensis					
TGCPNGCARPYMAEL<br>GIAVRAGHHCAQP	1582<br>1316	15<br>13	H_2O_2-stressed zebrafish larvae and leucocytes<br>7.8-125 μM<br>10-80 μM	- Dose-dependent *in-vitro* radicals scavenging activity (DPPH, ABTS, SARS and HRS)<br>↓ ROS production of leucocytes and larvae<br>↑ Antioxidant enzymes (SOD, CAT)<br>- Non-toxic to blood cells and zebrafish larvae	[93,104]
Arthrospira platensis					
GGGAFSGKDPTKVDR	1490	15	H_2O_2-stressed zebrafish larvae and MDCK cells<br>5-45 μM	- Dose-dependent *in-vitro* radicals scavenging activity (ABTS, SARS, HRS and NO)<br>↓ Oxidative stress and apoptosis of MDCK cells and zebrafish larvae<br>↑ Antioxidant enzymes (SOD, CAT)<br>↑ mRNA antioxidant enzymes gene expression (GPx, GST, GCS)<br>- Non-toxic to MDCK and zebrafish larvae	[95]

AAPH: 2,2′-Azobis(2-amidinopropane) dihydrochloride; ABTS: 2,2′-azino-bis(3-ethylbenzothiazoline-6-sulfonic acid); CAT: catalase; DPPH: 2,2-Diphenyl-1-picrylhydrazyl; FRAP: ferric reducing antioxidant power; GCS: glutamyl cysteine synthetase; GGT: gamma-glutamyl transferase; GPx: glutathione peroxidase; GST: glutathione S-transferase; H_2O_2: hydrogen peroxide; H9c2: embryonic rat cardiomyocytes cells; HaCaT: human immortal keratinocyte line; HepG2: human liver cancer cells; HO-1: heme oxgenase-1; HRS: hydrogen radical scavenging; HUVEC: human umbilical vein endothelial cells; L929: normal mouse fibroblasts; LPS: lipopolysaccharide; MAPK: mitogen-activated protein kinases; MDA: malondialdehyde; MDCK: Madin-Darby canine kidney cells.; MW: molecular weight; NF-κB: nuclear factor κB; NO: nitric oxide; Nrf2: nuclear erythroid 2-related factor 2; ORAC: oxygen radical antioxidant capacity; PCL: peptide chain length; SARS: superoxide anion radical scavenging; SH-SY5Y: Human neuroblastoma cells; SOD: superoxide dismutase; Vero cells: African green monkey kidney fibroblast

Table S2: Antihypertensive peptides derived from different microalgae species discovered in the past ten years

Peptide source/sequence	MW (Da)	PCL	IC_{50}/Model/ Dose	Inhibitory pattern/key findings	Ref.
Arthrospira platensis			**Cell-free ACE inhibition (IC_{50}) & molecular docking**		
ILLYR	676	5	10.54 µM	- Non-competitive inhibitor<br>- Interacted with ACE via hydrogen bonds, and bound to Cl2 binding site<br>- Relatively stable under gastrointestinal conditions (IC_{50} = 23.35 µM)	[124]
VTY<br>LGVP	381<br>384	3<br>4	23.39 µM<br>45.76 µM	- VTY interacted with ACE active pockets (S1′ & S2′) through hydrogen bonds, while LGVP formed fewer hydrogen bonds with S1 active pocket	[114]
IRDLDYY	957	7	1748 µM	- Interacted with ACE active pocket via hydrogen bonds<br>- Non-toxic to Vero and human dermal fibroblast cells	[85]
PTGNPLSP	781	8	1971.50 µM	- High stability under temperature (25-100°C), pH (3-11) and gastrointestinal digestion	[48]
Chlorella pyrenoidosa					
LKKAP<br>LVAKA<br>PGLRP	555<br>500<br>538	5<br>5<br>5	36.19 µM<br>26.66 µM<br>44.78 µM	- Non-competitive inhibitors<br>- Interacted with ACE active pockets S1, S2 and/or S′ via hydrogen bonds and salt bridges<br>- High stability under pH (pH 2-12) and temperature (20-100°C)<br>- High gastrointestinal stability and released new di/tri-peptides with low IC_{50} (AP=230 µM, VAK=85.43 µM, PGL=13.93 µM and RP=15.20 µM.	[20]
Chlorella sorokiniana					
VW<br>IW<br>LW<br>WV	303<br>317<br>317<br>303	2<br>2<br>2<br>2	580 nM<br>500 nM<br>1.11 µM<br>307.61 µM	- Potent ACE inhibitory activity, particularly with those containing tryptophan at the C-terminal	[51]
Isochrysis galbana					
YMGLDLK	839	7	36.10 µM	- Non-competitive inhibitor<br>- Highly stable under gastrointestinal digestive enzymes	[126]
Tetradesmus obliquus					
GPDRPKFLGPF<br>WYGPDRPKFL	1230<br>1278	11<br>10	5.73 µM<br>820 nM	- Exhibited a dose-dependent ACE inhibitory activity with low IC_{50}	[109]
Nannochloropsis oculata					
NKFPYTTQ<br>LVGADAHALGVICS<br>VVGAVGAADLL<br>GDVGLF<br>KGGGSGANGGRL<br>AGDVGFDPLGF<br>VYNKFPYTTQ<br>YANDLLCMPI	998<br>1325<br>984<br>606<br>1030<br>1094<br>1260<br>1152	8<br>14<br>11<br>6<br>12<br>11<br>10<br>10	1.00 µM<br>1.17 µM<br>1.01 µM<br>1.65 µM<br>481.64 µM<br>913.92 µM<br>793.39 µM<br>867.76 µM	- Most of the peptides showed strong ACE inhibitory activity with low IC_{50} of 1-1.65 µM	[128]
			**Cell/*in-vivo* models & molecular docking**		
Isochrysis zhanjiangensis					
FEIHCC	750	6	- Cell-free ACE inhibition (IC_{50}) 61.38 µM<br>- Cell model Ang II-induced HUVEC 10-100 µM	- Non-competitive inhibitor<br>- Interacted with ACE and VCAM-1 through hydrophobic interaction and hydrogen bonds, and bound to ACE with Cl2 binding site (Arg522)<br>↓ Ang II-induced vascular dysfunction (↓ICAM-1, VCAM-1, MCP-1, iNOS & ET-1) and ROS<br>↓ Inflammation and apoptosis through NF-κB, Nrf2, MAPK, and Akt signalling pathways<br>- Non-toxic to HUVEC	[127]
EMFGTSSET	988	9	- Cell-free ACE inhibition (IC_{50})	- Non-competitive inhibitor<br>- Strongly bound to ACE and VCAM-1 active pockets	[122]

			15.08 μM<br>- Cell model<br>Ang II-induced HUVEC<br>10-100 μM<br>- *In-vivo* SHR<br>10 mg/kg rat body weight	through hydrogen bonds, with major contribution from Ser<br>↓ Ang II-induced vascular dysfunction (↓ ROS, IL-8, IL-1β, TNF-α, iNOS, COX-2, ET-1, ATR1, ICAM-1 & VCAM-1)<br>↓ Ang II-induced inflammation and apoptosis through NF-κB, MAPK and Akt signalling pathways (↓ endothelial damage)<br>- Oral administration of the peptide induced significant reductions in systolic blood pressure, and the activity was maintained for 10 h, like the Captopril treatment at 10 mg/kg<br>Non-toxic to HUVEC	
***Arthrospira* sp.**					
TVLYEH<br>LQAGGLF	760<br>704	6<br>7	- Cell-free ACE inhibition (IC_{50})<br>2.88 μM<br>66.83 μM<br>- *In-vivo* SHR<br>10-20 mg/kg rat body weight	- Competitive inhibitors<br>- Strong binding affinity to ACE active pockets through hydrogen bonds, hydrophobic interactions and electrostatic forces<br>- Stable under pH (2-12) and temperatures (0-100°C)<br>- TVLYEH was more stable under gastrointestinal digestion<br>- Oral administration significantly decreased SBP and DBP<br>- Long-lasting blood pressure reduction until 8 hours after oral administration, while Captopril maintained its effect within 2 hours	[83]
TMEPGKP	759	7	- Cell-free ACE inhibition (IC_{50})<br>132 μM<br>- Cell model<br>Ang II-induced HUVEC<br>62.5-250 μM	- Non-competitive inhibitor<br>- Interacted with ACE and Ang II via hydrogen and van der Waals interactions<br>↓ Ang II-induced vascular dysfunction (↓iNOS & ET-1) and ROS<br>↓ MAPK pathway<br>- Non-toxic to HUVEC	[123]
Arthrospira platensis					
IQP<br>VEP	356<br>440	3<br>3	- Cell-free ACE inhibition (IC_{50})<br>5.77 μM<br>27.36 μM<br>- *In-vivo* SHR<br>10 mg/kg rat body weight/day	- Non-competitive inhibitors<br>- Long-term oral intake (8 weeks) significantly decreased SBP, DBP, left ventricular mass index and right ventricular mass index<br>- Regulated the main components of RAAS in myocardium and kidney (↓ ACE, Ang II & ATR1) ACE-Ang II-ATR1 axis<br>↑ ATR2, ACE2, Ang-(1-7) and Mas receptor (ACE2-Ang-(1-7)-Mas axis)<br>- Non-toxic to SHR	[119,120]
GIVAGDVTPI	941	10	- *In-vivo* SHR<br>10 mg/kg rat body weight	- Oral administration significantly decreased SBP (~43 mmHg) between 2 and 8 hours<br>- NO-dependent mechanism by activating PI3K/AKT/eNOS pathways	[125]
Chlorella pyrenoidosa					
LRAKA	557	5	- Cell-free ACE inhibition (IC_{50})<br>350 nM<br>- *In-vivo* SHR<br>20 mg/kg rat body weight	- Modified peptide from its precursor peptide (LVAKA) based on alanine scanning and saturated mutations (by substituting Val2 with Arg)<br>- The peptide showed a potent ACE inhibitory activity with a very low IC_{50} (nanomolar), ranking it in the top 1% ACE-inhibitory peptides<br>- Reduced SBP (-20.50 mmHg) and DBP (-17.70 mmHg) in SHR within 12 hours at 20 mg/kg body weight.	[20]
Chlorella vulgaris					
TTW<br>VHW	406<br>441	3<br>3	- Cell-free ACE inhibition (IC_{50})<br>610 nM<br>910 nM<br>- *In-vivo* SHR<br>5 mg/kg rat body weight	- Non-competitive inhibitors<br>- Strong binding to ACE active pockets through hydrogen bonds<br>- Stable under gastrointestinal digestion conditions and ACE hydrolysis<br>- Oral administration significantly decreased the SBP and DBP, comparable to Lisinopril	[86]

ACE: angiotensin-converting enzyme; Akt: serine/threonine kinase; Ang II: Angiotensin II; COX-2: cyclooxygenase-2; DBP: diastolic blood pressure; ET-1: endothelin-1; ICAM-1: intracellular adhesion molecule 1; IL-1β: interleukin 1β; IL-8: interleukin 8; iNOS: inducible nitric oxide synthase; MAPK: mitogen-activated protein kinases; MW: molecular weight; NF-κB: nuclear factor κB; NO: nitric oxide; Nrf2: nuclear erythroid 2-related factor 2; PCL: peptide chain length; SBP: systolic blood pressure; SHR: spontaneously hypertensive rats; TNF-α: tumour necrosis factor-α; VCAM-1: vascular cell adhesion molecule 1.

Table S3: Antidiabetic and anti-obesity peptides derived from microalgae discovered in the past ten years

Peptide source/ sequence	MW (Da)	PCL	Inhibitory of diabetic-related enzymes (IC_{50}/ Activity)			Inhibition pattern/ Mechanism/Key findings	Ref.
**Antidiabetic peptides**							
Chlorella vulgaris			**DPP-IV**	**α-glucosidase**	**α-amylase**		
VPW<br>IPR<br>VPA<br>IPL	400<br>384<br>285<br>341	3<br>3<br>3<br>3	348.60 μM<br>376.60 μM<br>503.50 μM<br>466.00 μM	-	-	- Competitive inhibitors (VPW & IPR)<br>- VPW and IPR bound with DPP-IV active pockets S1 or S2 through hydrogen bonds, Van der Waals and hydrophobic interactions<br>- High gastrointestinal stability, and good DPP-IV inhibitory in mouse serum (IC_{50}=2.5-3.5 mM)	[140]
***Arthrospira* sp.**							
GPNYASSER<br>TPLGMVSML<br>SLPLGGN<br>SFVAGTM<br>VMAPPAP<br>GIPISTFIG<br>ASGGSMP	980<br>948<br>656<br>711<br>681<br>904<br>605	9<br>9<br>7<br>7<br>7<br>9<br>7	358 μM<br>397 μM<br>415 μM<br>808 μM<br>953 μM<br>>5000 μM<br>>5000 μM	-	-	- Inhibited the activity of DPP-IV in a concentration-dependent manner, except for GIPISTFIG and ASGGSMP which showed the highest IC_{50} of >5000 μM	[28]
Chlorella sorokiniana							
VW<br>IW<br>LW<br>WV	303<br>317<br>317<br>303	2<br>2<br>2<br>2	27.70%**<br>15.5%**<br>23.80%**<br>12.70%**	26.70%**<br>33.80%**<br>27.90%**<br>57.70%**	-	- Relatively higher α-glucosidase inhibitory activity than DPP-IV<br>- Interacted with α-glucosidase at the pocket near the N-terminal β-sheet domain and behind the β-strands of the catalytic domain<br>- VW, IW, LW were located within the catalytic domain (Ser630, Asp708, His740) of DPP-IV<br>- WV was positioned far from the catalytic site of DPP-IV, which had the lowest DPP-IV activity	[84]
Arthrospira platensis							
GVPMPNK<br>RNPFVFAPTLLTVAAR<br>LRSELAAWSR	741<br>1772<br>1187	7<br>16<br>10	259.51 μM<br>102.26 μM<br>140.94 μM	204.45 μM<br>92.83 μM<br>113.06 μM	318.76 μM<br>608.13 μM<br>264.20 μM	- Strong concentration-dependent inhibitory activity<br>- Their inhibitory activity (%) towards α-amylase and DPP-IV was almost similar with acarbose and sitagliptin, while 2-fold higher than acarbose against α-glucosidase	[91]
**Anti-obesity peptides**			**Model/Effective concentration/Dose**				
Chlorella pyrenoidosa			**Cell-free**				
SISISVAGGGR<br>KQTALVELVK<br>KNGAPAEK<br>SRQLTLYPGAER<br>LLVVYPWTQR<br>SDDPHTFGQGTK	1003<br>1128<br>814<br>1390<br>1274<br>1289	11<br>10<br>8<br>12<br>10<br>12	- Pancreatic lipase inhibition<br>- Bile salts adsorption capacity<br>- Cholesterol-lowering capacity<br>- Gut modulation in high cholesterol colonic fermentation model<br>- 5-20 mg/mL			- SISISVAGGGR (99.49%) and LLVVYPWTQR (63.03%) had the highest pancreatic lipase inhibitory activity<br>- SISISVAGGGR (10.49%) had the highest bile salts adsorption capacity<br>- SISISVAGGGR (28.99%) and SRQLTLYPGAER (23.60%) had the highest cholesterol-lowering effect and gastrointestinal stability<br>- SISISVAGGGR modulated gut microbiota: ↑beneficial bacteria (*Lactobacillus* & *Akkermansia*) and ↓harmful bacteria (*Escherichia-Shigella*)	[54]
Arthrospira platensis			**Cell model**				
CANPHELPNK	1122	10	- Lipid inhibition in 3T3-L1 cells			↓ preadipocytes differentiation	[57]

NPVWKRK<br>NALKCCHSCPA<br>LNNPSVCDCDCMMK<br>AAR	927<br>1146<br>1871	7<br>11<br>17	- 0.60-2.00 mg/mL	(32-60%) at 0.2 mg/mL<br>- CANPHELPNK and NPVWKRK reduced the accumulation of TG by 19.50 and 23.70%, at 0.60 mg/mL<br>- Non-toxic to L-O2 cells	
Chlorella pyrenoidosa			**Cell model**		
LLVVYPWTQR	1274	10	- Pancreatic lipase inhibition<br>- Lipid inhibition in 3T3-L1 cells<br>- 0.20-0.60 mg/mL	- Interacted with pancreatic lipase through hydrogen bonds at the catalytic sites (Ser153, Asp177 and His 264)<br>↓ Pancreatic lipase by 47.95%<br>↓ TG by 27.90% at 0.60 mg/mL, comparable to simvastatin (24.10%)<br>↓ Fat accumulation and fatty acid synthesis in adipocyte cells by activating the ↑AMPK pathway and ↓ adipogenic-specific proteins (C/EBPα, SREBP-1c and PPARγ)	[56]
Chlorella pyrenoidosa			***In-vivo***		
SISISVAGGGR	1003	11	- High-fat diet induced obesity in C57BL/6J male mice<br>- 100-600 mg/kg mice body weight/d	↓ Body weight, epididymal fat weight, heart weight index, hepatic lipid accumulation and prevented liver inflammation<br>↓ Fasting glucose level and improved insulin resistance<br>↓ Serum levels of TG and LDL, while ↑HDL<br>↓ Firmicutes/Bacteroidetes ratio<br>↓ Obesogenic and proinflammatory bacteria such as *Acetatifactor*, *Dorea* and *Ruminococcus gnavus*<br>↑ Beneficial bacteria such as *Bacteroides*, *Parabacteroides* and *Alistipes*<br>↑ Regulation of pathways involved in carbohydrates metabolism, amino acids metabolism and membrane transport<br>- The highest concentration has better anti-obesity effects	[54,55]
Haematococcus pluvialis			***In-vivo***		
KFTPAP	659	6	- Wild-type *C. elegans* strain N2 nematodes<br>- 100 µM	↓ Fat content (12.97%), TG (19.62%), lipid droplets (22.75%)<br>↑ Ratio of oleic acid (C18:1n9) to stearic acid (C18:0), promoting the desaturation of C18:0 to C18:1n9<br>↑ Energy expenditure by increasing locomotor activity and shortening the defecation cycle<br>↑ fat-6 and fat-7 genes (Fat-6/Fat-7 Pathway), which are involved in lipid metabolism<br>↑ Activated NHR-49/PPARα pathway, enhancing fatty acid β-oxidation and promoting lipolysis<br>↑ Activated AAK-2/AMPK pathway, which is involved in regulating energy balance and lipid mobilisation	[147]

AAK-2: AMP-activated protein kinase catalytic subunit alpha-2; AMPK: adenosine monophosphate-activated protein kinase; C/EBPα: CCAAT/enhancer binding protein alpha; DPP-IV: dipeptidyl peptidase-4; HDL: high-density lipoprotein; LDL: low-density lipoprotein; L-

O2: normal liver cells; NHR-49; nuclear hormone receptor 49; PPARγ: peroxisome proliferator-activated receptor gamma; SREBP-1c: sterol regulatory element-binding protein 1c; TG: triglyceride.
** Activity was tested at peptide concentration of 0.25 µg/mL.

Table S4: Anti-ageing peptides derived from different microalgae species discovered in the past ten years

Peptide source/ sequence	MW (Da)	PCL	Model/Dose	Inhibition mechanism/Key findings	Ref.
			**Cell-free**		
Arthrospira platensis					
DER	418	3	-Tyrosinase (diphenolase) inhibitory activity	- Interacted with tyrosinase through hydrogen bonds with the key residues His85, His244, His259 and Asn260<br>- Strong inhibitory activity (IC_{50} = 1.04 mM)	[92]
			**Cell model**		
Arthrospira platensis					
AFGRFR<br>MAACLR<br>RCLNGRL<br>RYVTYAVF<br>SPSWY<br>GRF<br>AADQRGKDKCARDIGY	752<br>663<br>831<br>1018<br>638<br>378<br>1766	6<br>6<br>7<br>8<br>5<br>3<br>16	- *In-vitro* tyrosinase (monophenolase & diphenolase) inhibitory activity<br>- Melanoma cell: tyrosinase inhibitory activity and melanin production	- SPSWY (IC_{50}=12.1 µM) and MAACLR (86.9 µM) showed the highest monophenolase inhibitory activity<br>- Only MAACLR and RCLNGRL showed both monophenolase (IC_{50} = 86.9 µM & 116.20 µM) and diphenolase (IC_{50}=990.3 & 1069.8 µM) inhibitory activity<br>- Only SPSWY, GRF and AADQRGKDKCARDIGY had cellular diphenolase inhibitory (IC_{50}=34.2-363.2 µM)<br>- All the peptides reduced cellular melanin synthesis by 47.05-75.54% at 200 µg/mL, except RYVTYAVF which increased melanin synthesis up to 3-fold (potential pigmentation agent)<br>- Non-toxic to melanoma cells up to 200 µM	[155]
GMCCSR	655	6	- UVB (80 mJ/m^2)-induced HSF<br>15.20 µM	↑ Proliferation of human skin fibroblasts and restored collagen production by 16.5% (similar to original level) after UVB irradiation<br>- Non-toxic to human erythrocyte and normal mouse fibroblasts	[102]
Isochrysis zhanjiangensis					
DAPTMGY	753	7	- UVB (40 mJ/m^2)-Irradiated HaCaT cells<br>20-100 µM	↑ Cell viability after UVB exposure and anti-apoptotic protein Bcl-2<br>↓ Pro-apoptotic proteins (Bax, caspase-8/-3) and intracellular ROS<br>↑ Antioxidant enzymes (SOD, CAT and GPx)<br>↓ Collagen-degrading enzymes MMP-1 and MMP-3<br>↑ Collagen synthesis via increased procollagen I expression<br>↓ Suppressed photoageing by inhibiting phosphorylation of MAPK pathway proteins (ERK, JNK and p38), AP-1 and NF-κB signalling pathway (↓ p-p65 & p-IκB)<br>- Non-toxic to human keratinocyte cells up to 200 µM	[152]
AYAPE	549	5	- UVB (40 mJ/m^2)-irradiated HaCaT cells<br>10-50 µM<br>- H_2O_2 (600 µM)-induced senescence in BJ cells<br>10-50 µM	↑ Viability of stressed human keratinocyte and dermal fibroblasts<br>- Non-toxic to human keratinocyte and dermal fibroblasts cells up to 150 µM<br>UVB-induced human keratinocyte cells (HaCaT):<br>↓ Intracellular ROS and DNA damage<br>↓ Collagen-degrading enzyme MMP-1<br>↑ Anti-apoptotic effect (↑Bcl/Bax ratio, ↓nuclear P53, ↓cleaved caspase-3/-9)<br>↓ MAPK/AP-1 pathway (↓ p-p38, p-ERK, p-JNK, p-c-Jun)<br>↓ NF-κB signalling (↓ p-p65, iNOS, COX-2)<br>Human dermal fibroblast cells (BJ):<br>↓ Senescence markers (↓p53, p21 & p16) and p-Rb phosphorylation (↓AKT activation)<br>↓ Cellular ageing (fewer β-galactosidase-positive cells)	[153]
EMFGTSSET	988	9	- UVB (40 mJ/m^2)-irradiated	↑ Viability of stressed human keratinocyte and dermal fibroblasts<br>- Non-toxic to keratinocyte (200 µM) and dermal fibroblasts	[151]

			HaCaT cells<br>50-200 μM<br>- H_2O_2 (600 μM)-induced senescence in in BJ cells<br>50-200 μM	cells (300 μM)<br>- Comparable anti-ageing effect to vitamin C<br>UVB-induced human keratinocyte cells (HaCaT):<br>↓ ROS through ↑ expression of Nrf2/HO-1 signalling pathway<br>↑ Autophagosomes, lysosomes and autophagy-related proteins (↑LC3, Beclin 1, ATG5-ATG12) by ↑expression of phosphorylated AMPK and ↓phosphorylated mTOR (mTOR/AMPK signalling pathway)<br>↑ Mitochondrial potential (↑Bcl-2/Bax ratio, ↓p53, ↓caspase-3/-9)<br>Human dermal fibroblast cells (BJ):<br>- Regulated senescence-related factors (↓ p53, ↓ p21, ↓ p26, ↑ phosphorylated Rb)<br>↑ Collagen generation: ↑TGF-β1 and ↓phosphorylated Smad2/3 (TGF-β1/Smad pathway)	
			***In-vivo* model**		
Arthrospira platensis					
Ac-GMCCSR-NH_2	655	6	- UVB (35 mJ/m^2)-induced HaCaT cell model<br>10 mg/mL<br>- UVB (60 mJ/m^2)-induced mice dorsal skin<br>10 mg/mL	↑ Proliferation of UVB-irradiated HaCaT cells<br>- Normalised epidermal thickness and restored skin moisture; ↓ inflammation and vacuolar degeneration in irradiated mice<br>↑ Antioxidant enzyme activities (↑ CAT, GPx) and ↓ collagen-degrading enzymes (MMP-1, MMP-3) and ↓ MDA level in skin tissue of mice<br>- Proteomics revealed regulation of 60 proteins and involvement of HIF-1, glycolysis, and keratinisation pathways<br>- Non-toxic to HaCaT cells and safe to mice	[154]
***Synechococcus* sp. VDW**					
AILESYSAGKTK	1267	12	- Cell-free tyrosinase inhibitory activity (monophenolase & diphenolase)<br>- Melanoma cells model<br>6.25-100 μM<br>- Zebrafish embryos<br>6.25-100 μM	- Strong cell-free monophenolase (IC_{50}=489.71 μM) and diphenolase (IC_{50}=765.57 μM) inhibitory activity<br>- Competitively inhibited tyrosinase through binding with the active site via hydrogen bonds (His244, Arg268, Cys83) Pi-alkyl (His263, Phe264, Val248) and Van der Waals interactions<br>↓ Melanin content in melanoma cells and zebrafish embryos in a concentration-dependent manner<br>↓ Melanogenesis-related genes (TYR, TRP-1 & TRP-2)<br>↑ MITF<br>- Non-toxic to melanoma cells up to 400 μM<br>- Safe to zebrafish embryos up to 100 μM	[157]
Haematococcus pluvialis					
KFTPAP	659	6	- Wild-type *C. elegans* strain N2 nematodes (normal and stress-induced ageing models)<br>100 μM	↑ Lifespan of nematodes under normal, oxidative and thermal stress conditions<br>↑ physiological functions of nematodes by ↓ ageing pigment (lipofuscin) and ↑ locomotion and pharyngeal pumping activity without impairing reproduction<br>↑ SOD and CAT activity and ↓ MDA level<br>↑ Genes expression related to stress resistance (skn-1, hsp-16.2) and antioxidant activity (sod-3 and ctl-2) and IIS pathway (daf-16, daf-2, ins-18 and sir-2.1)<br>- IIS pathway was critical to the peptide's anti-ageing mechanism, through the regulation of subcellular localisation of DAF-16	[156]

AMPK: adenosine 5′-monophosphate (AMP)-activated protein kinase; ATG12: autophagy-related 12; ATG5: autophagy-related 5; B16-F10: melanoma cells; Bcl-2: B-cell lymphoma 2; Beclin 1: Bcl-2 interacting protein 1; BJ cells: human dermal fibroblasts; CAT: catalase; GPx: glutathione peroxidase; HaCaT cells: human keratinocyte; HO-1: heme oxgenase-1; HSF: human skin fibroblasts; IIS: insulin/insulin-like growth factor signalling; LC3: microtubule-associated protein 1 light chain 3; MAPK: mitogen-activated protein kinases; MDA: malondialdehyde; MITF: microphthalmia-associated transcription factor; MMP: metalloproteinases; mTOR: mammalian target of rapamycin; NF-κB: nuclear factor κB; Nrf2: nuclear erythroid 2-related factor 2; SOD: superoxide dismutase; TGF-β1: transforming growth factor β1; TRP: tyrosinase-related protein; TYR: tyrosinase; UVB: ultraviolet B radiation.
